# Genetic diversity of strawberry germplasm using metabolomic biomarkers

**DOI:** 10.1038/s41598-018-32212-9

**Published:** 2018-09-26

**Authors:** José G. Vallarino, Francisco de Abreu e Lima, Carmen Soria, Hao Tong, Delphine M. Pott, Lothar Willmitzer, Alisdair R. Fernie, Zoran Nikoloski, Sonia Osorio

**Affiliations:** 1 Instituto de Hortofruticultura Subtropical y Mediterránea “La Mayora” – University of Malaga- Consejo Superior de Investigaciones Científicas (IHSM-UMA-CSIC), Department of Molecular Biology and Biochemistry; Campus de Teatinos, 29071 Málaga, Spain; 2 Max-Planck-Institut für Molekulare Pflanzenphysiologie, Am Mühlenberg 1, 14476 Potsdam-Golm, Germany; 3 Instituto Andaluz de Investigación y Formación Agraria y Pesquera (IFAPA), Centro de Churriana, Málaga, Spain

## Abstract

High-throughput metabolomics technologies can provide the quantification of metabolites levels across various biological processes in different tissues, organs and species, allowing the identification of genes underpinning these complex traits. Information about changes of metabolites during strawberry development and ripening processes is key to aiding the development of new approaches to improve fruit attributes. We used network-based methods and multivariate statistical approaches to characterize and investigate variation in the primary and secondary metabolism of seven domesticated and seven wild strawberry fruit accessions at three different fruit development and ripening stages. Our results demonstrated that *Fragaria* sub-species can be identified solely based on the gathered metabolic profiles. We also showed that domesticated accessions displayed highly similar metabolic changes due to shared domestication history. Differences between domesticated and wild accessions were detected at the level of metabolite associations which served to rank metabolites whose regulation was mostly altered in the process of domestication. The discovery of comprehensive metabolic variation among strawberry accessions offers opportunities to probe into the genetic basis of variation, providing insights into the pathways to relate metabolic variation with important traits.

## Introduction

Modern domesticated ripe strawberry (*Fragaria x ananassa* Duch.) fruit is characterized by its large size^1^, red color^2^, distinct aroma^3^, sweet fruity flavor^4^, and reduced firmness^5^. The flesh of the strawberry is a swollen receptacle, a false fruit, and the seeds (achenes) are the true fruit^6^. During the early stages of strawberry fruit development, auxin, synthetized in the achenes, promotes fruit growth^7,8^. Receptacle growth follows either a single or a double sigmoid curve, depending on the cultivar, and involves gains in diameter and fresh weight. This change is mainly a result of cell enlargement; however, cell division accounts for 15–20% of the total growth^9,10^.

Strawberry growth and ripening are complex developmental processes that involve many events. The latter involves biochemical and physiological changes that occur in the final stages of fruit development and maturation, such as cell wall modification, starch conversion to sugars, anthocyanin pigments biosynthesis, flavor and aromatic volatile accumulation, as well as an increase in polyphenols and other antioxidant compounds^11,12^. During strawberry fruit development sucrose is continually imported from photosynthetic tissue, which initiates a complex network of primary and secondary metabolism specific to ripening strawberry fruit^13^. Metabolic profiling indicates an accumulation of sugars, organic acids, and fatty acids as well as the consumption of amino acids during fruit development^13,14^. The content of sugars, organic acids, amino acids as well as other soluble component play a significant role in the overall flavor of fruit^15,16^.

Compared with other fruits, strawberries have a high *in vitro* antioxidant activity, which can be largely attributed to some phytochemicals, including: ascorbic acid, phenolics, anthocyanins, and other flavonoid compounds^17,18^. Polyphenols compounds display the most noticeable metabolic changes during strawberry fruit development, and the degree of ripeness considerably affects both their absolute levels and relative proportions. In general, the phenolic acid concentrations decrease during ripening, while the anthocyanin concentrations increase^13,19^. During early stages, flavonoids, mainly condensed tannins, accumulate to high levels and provide immature fruit an astringent flavor^13,20–22^. When the fruit starts to ripe, other flavonoids, such as anthocyanins, cinnamic and coumaric acid derivatives, and flavonols accumulate to high levels^13,22,23^, all of them with high antioxidant capacity^24–26^.

It is known that the genotype has an effect on the metabolic composition of strawberry, which has a direct impact on nutritional and organoleptic quality^3,27–30^, although only a few genotypes have been well characterized for these important features. For example, the aroma of wild strawberries is very pleasant and more herbaceous than that of domesticated varieties. Therefore, wild type berries could be a more desirable raw material for the food industry^31^. However, the low productivity and varying annual yields of wild strawberries have restricted their cultivation.

The potential for breeding of cultivars that are more nutritious, better-tasting and more resistant to biotic/ abiotic stress depends to an extent of the variability and heritability of the bioactive compounds. The biotechnological approach is also an option to supplement this improvement, via modification of specific biosynthetic pathways^32–38^. However, the success of both breeding and biotechnological approaches is dependent knowledge of the source of the most useful wild and domesticated genetic diversity to be used in genetic and genomic studies. Utilizing wild species as accessions of the progenitor as sources of novel traits are valued by strawberry breeders^39^. Furthermore, previous investigations showed improvements in fruit quality in breeding material that originated from *Fragaria virginiana* ssp. *glauca* (FVG) in inter-species crosses^24,32,40,41^. In their earlier study^24^, the authors showed that the fruit of *F*. *virginiana* accessions have significantly higher antioxidant capacity, total phenol and anthocyanins contents than fruit from different lines of *F*. *chiloensis* and *F*. *x ananassa*.

Strawberry breeding has become an area of substantial economic importance, mainly as a result of the royalties paid for commercial protected cultivars. Current methods for accurate detection of erroneous or illegal propagation are time-consuming and expensive because they involve the cultivation and morphological characterization of controversial cultivars for one to two years under controlled conditions.

Metabolomic approaches, including those utilizing Gas Chromatography-Mass Spectrometry (GC-MS) and Liquid Chromatography-Mass Spectrometry (LC-MS) allow the single-experiment profiling and quantification of numerous metabolites that are generally conserved across the kingdoms of life^42^. Metabolomics can take a snapshot of the current biochemical status, useful to compare varieties and evaluate changes in metabolic pools^13,43–47^. These metabolic phenotypes can be used in comparative analysis of metabolic processes across accessions to obtain temporal aspects of biochemical regulation. These large datasets are analyzed with multivariate statistical analysis aimed at determining biological components that show differential behavior under various scenarios within a single species. While the field of using molecular markers for breeding has experimented a revolution due to the advent of next-generation sequencing technologies^48–51^. Evolutionary metabolomics studies including wild and domesticated accessions have been carried out for few selected species^52^. The aim of this work is to provide a detailed comparative analysis of wild and domesticated accessions with respect to primary and secondary metabolism in fruit ripening stages of 14 *Fragaria* accessions (seven domesticated and seven wild accessions) and to determine if data from metabolomics technologies have the power to distinguish between two accession types. In addition, if the latter is feasible, we are also interested in identifying the metabolites which contribute most to the distinction between the two groups of accessions. Thus, the selected metabolites can be used either as markers for selection, certification of different species or for the physiological evaluation of plant genotypes for breeding new strawberry cultivars.

## Results

### Metabolic profiling during domesticated and wild strawberry fruits development and ripening

**Figure 1:**
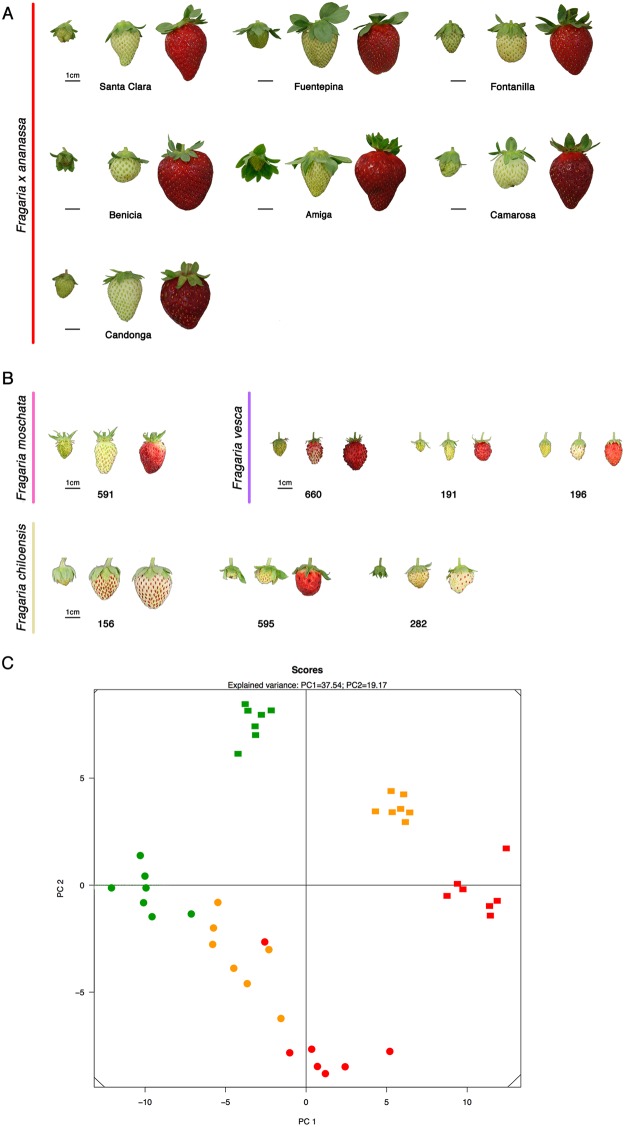
(**A**,**B**) Anatomic structures of domesticated and wild strawberry accessions. Bars = 1 cm. (**C**) Principal component analysis (PCA) of strawberry accessions based on primary and secondary metabolite profiles. Score plots of the PCA, domesticated (squares) and wild (circles) strawberry accessions. Green symbols, green stage; orange symbols, white stage; red symbols, red stage.

To assess the metabolic profiles reflecting the interplay between primary and secondary metabolisms in the fruit of domesticated as well as wild strawberry accessions, we selected three stages of ripening (Methods). For the comparative analysis of metabolite profiles, we used the profiles of 43 primary metabolites, from GC-MS technology, and 87 polar secondary metabolites, from UPLC-Orbitrap-MS/MS in seven domesticated and seven wild strawberry accessions over three different ripening stages. First, we examined the quality of the normalized data sets by principal components analysis (PCAs) on the combined metabolic profiles from domesticated and wild accessions together (Fig. 1).

The major diagonal passing through (5, 5) and (−5, −5) separates the green from the other stages, whereas the diagonal passing through (−5, 5) and (5, −5) separates the domesticated from wild strawberry accessions (Fig. 1). This analysis highlights a clear metabolic shift among the first stage of ripening (green, G) to later ripening stages (white, W and red, R) when the fruit reaches its final size and color (Fig. 1). Moreover, separate PCA for the metabolic profiles from GC-MS and UPLC-Orbitrap-MS/MS reveals similar pattern (Supplementary Fig. S1).

The dendrogram produced by hierarchical cluster analysis (HCA) of the strawberry accessions based on the variation in 43 primary metabolites and 87 polar secondary metabolites (Supplementary Fig. S2A,B, respectively), confirmed the results observed with PCA and provided a more detailed view on the relationships between accessions. HCA showed a clear separation between domesticated and wild accessions, confirming the PCA results for primary metabolites (Supplementary Fig. S2A). However, HCA for polar secondary metabolites revealed some more dynamic changes when comparing wild and domesticated strawberry accession (Supplementary Fig. S2B). Moreover, structurally related metabolites derived from the same metabolic pathway clustered together as previously shown^53^.

### STATIS analysis based on the metabolome reveals patterns of association between accessions and ripening stages

In the following, we investigated how similar are the entire data sets of metabolite relative abundance between different accessions. This allows us to arrive at a compromise between the different data sets, which provided additional insights in the relationship between the accessions. To this end we applied analysis based on STATIS which, unlike PCA, allows simultaneous investigation of multiple tables (i.e., data sets). In our analysis, we used altogether *K* = 14 tables that correspond to the seven domesticated and seven wild strawberry accessions, where the observations correspond to 130 metabolites (primary and polar secondary metabolites) over three ripening stages in each accession. The compromise of the tables is based on the R_v_ coefficients determined for every pair of tables. The Rv coefficient takes values between 0 and 1, and small values of the coefficient indicate large differences between the data sets. The compromise allows us to make statements about the concordance (i.e. similarity) between each pair of tables.

**Figure 2:**
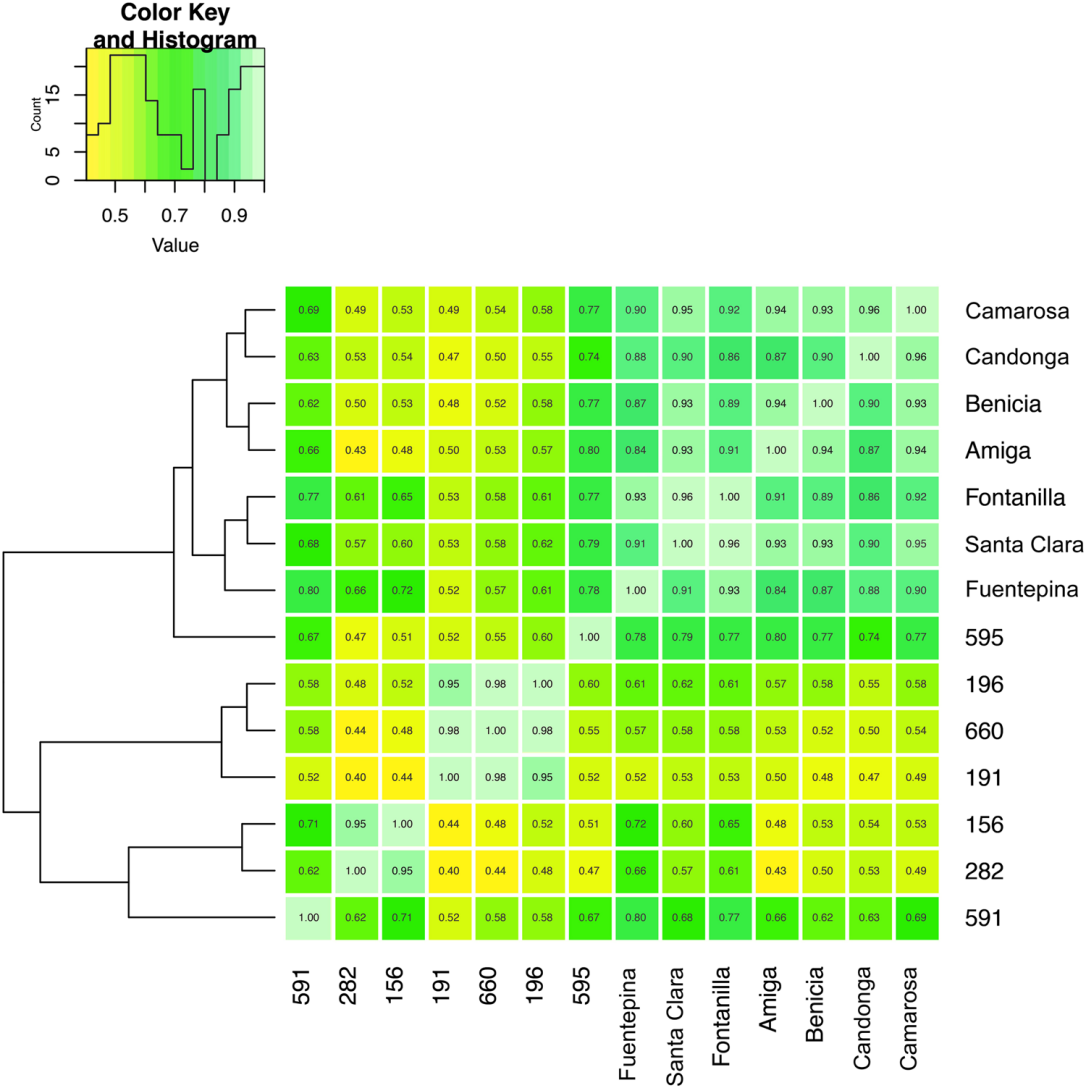
Heatmap and hierarchical clustering illustration the similarity of the 14 data tables analyzed (primary and secondary metabolites). The pairwise similarity is derived using the Rv coefficient of the corresponding cross-product matrices obtained for each condition. A value of 1 for the Rv coefficient corresponds to equivalence (light green), while a value of 0 indicates complete dissimilarity (yellow).

Figure 2 includes the heatmap that visualizes the concordance between accessions for the joint consideration of primary and secondary metabolites. The heatmap is a first indication that the investigated three ripening stages between domesticated and wild accessions are highly dissimilar. Moreover, Fig. 2 suggests that for the measured metabolites, there is a high similarity between all domesticated strawberry fruits, but this is not the case for the wild accessions. Finally, Supplementary Fig. S3 suggests that for the analyzed secondary metabolites, there is similarity between subspecies (*F*. *vesca*; 196, 191, 660, and *F*. *chiloensis*; 156 and 282).

**Figure 3:**
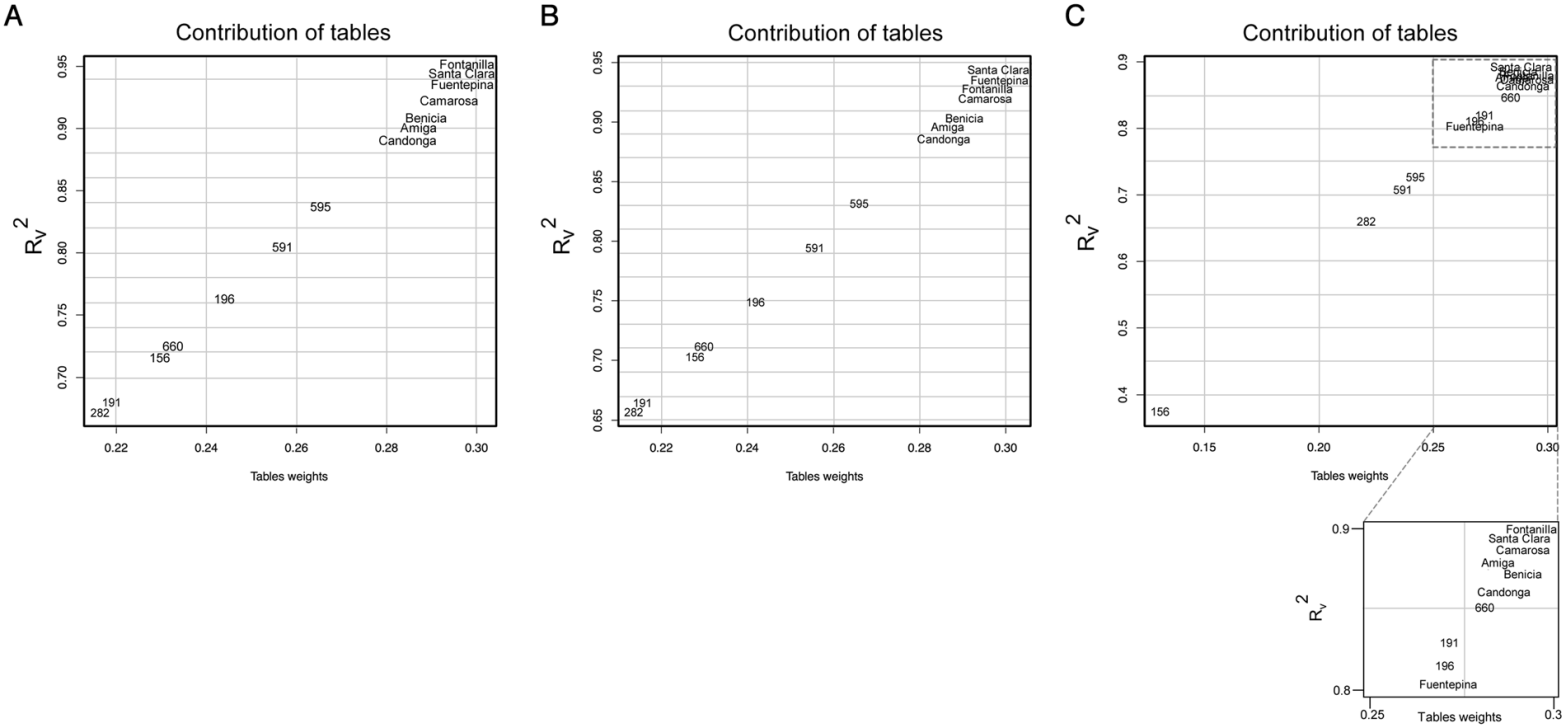
STATIS analysis of the importance of data tables. (**A**) Contribution of tables obtained for all 14 data tables used in STATIS for 130 analyzed metabolites. The x-axis denotes the table weights and y-axes displays the R_v_^2^ coefficient allowing assess the influence of tables on the resulting compromise. (**B**,**C**) Same analysis as in “a” but considering only secondary metabolites (87 metabolites) and primary metabolites (43 metabolites), respectively.

We next investigated the extent to which data sets from different accessions determine the compromise *S* (see Methods). To visualize the contribution of tables to the compromise we plotted the weight of each table on the x-axis and the similarity between the compromise and each individual table (per R_v_2 coefficient) on the y-axis (Fig. 3A). We found that domesticated strawberry fruits, the table weights (and the R_v_2 coefficients) are concentrated in the top right corner and, therefore, most affect that compromise; in contrast, those of wild strawberry are spread out (Fig. 3A). This indicates that the metabolites changes in the domesticated strawberry dominate the compromise of the entire data set. Moreover, the wild accessions, 282, 156 (two *F*. *chiloensis* accessions, 191 and 660, two *F*. *vesca* accessions) exhibit the smallest effect, manifested in the low R_v_2 coefficients and weights of tables. What is apparent from the contribution of each table to the compromise is that the domesticated accessions are very similar to each other, in contrast to the wild accessions, with the exception of 595. This finding may partly be due to the fact that *F*. *chiloensis* red accession was likely contributed as one parental of *F*. *x anannassa* strawberry (domesticated) accessions^54^. Therefore, this analysis points at a greater variability of the metabolome in wild compared to domesticated accessions.

Since, in this analysis, we consider two metabolites data sets, from primary and secondary metabolites, we next ask if the obtained compromise differ when the two classes of metabolites are analyzed separately. To this end, we determined the contribution of each table based on each class of metabolites, separately. As show in Fig. 3B, when considering only secondary metabolites, the compromise remained largely unaffected. However, this scenario changes when considering primary metabolites (Fig. 3C). This result shows that primary metabolic changes across ripening between domesticated and wild strawberry accessions are very similar, and that differences between the wild and domesticated accessions across the ripening stages are primarily due to the secondary metabolites. The exception is the wild accession 156 which stands out from the domesticated accessions with respect to primary metabolites.

**Figure 4:**
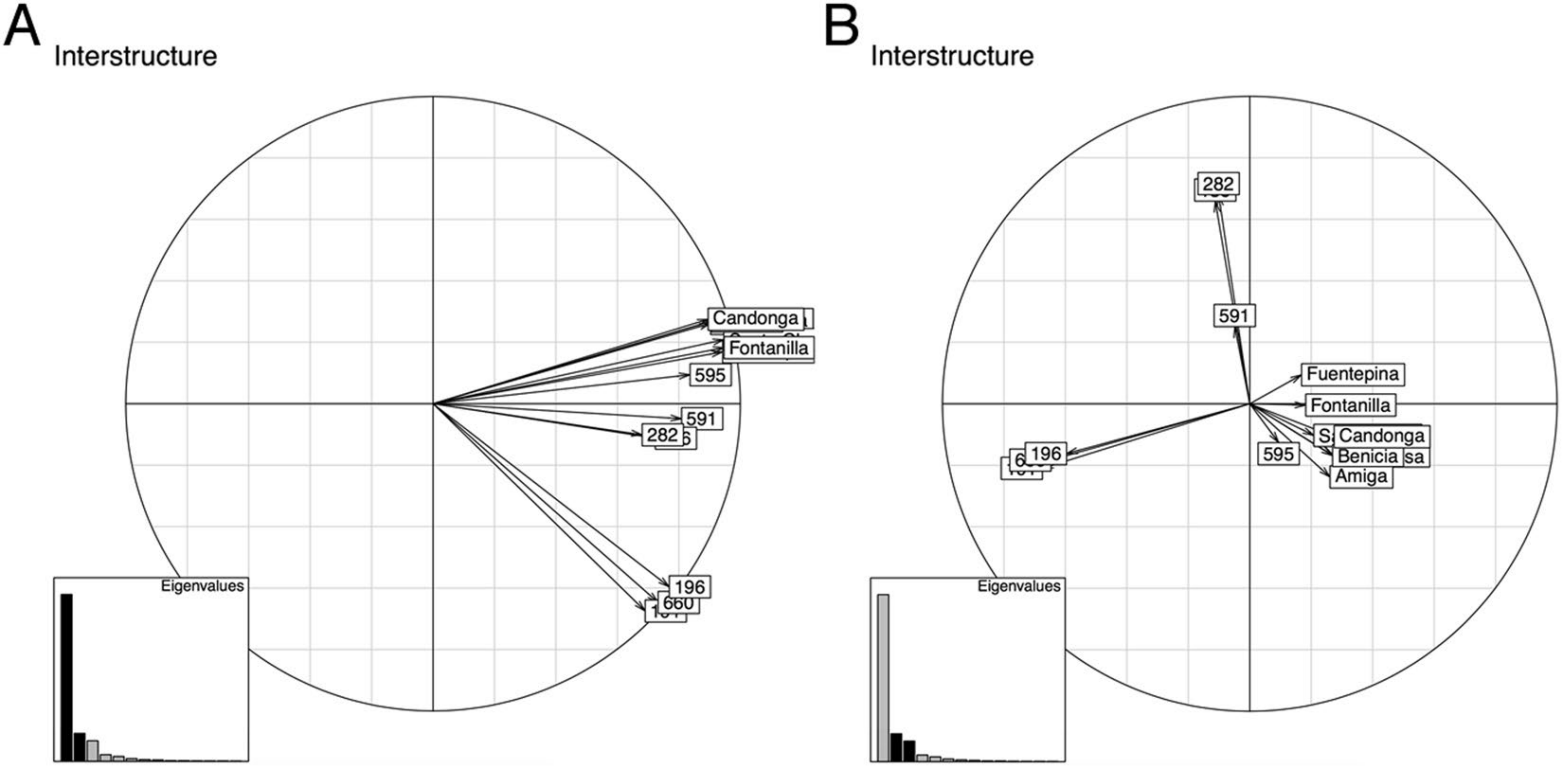
Interstructure of data tables. Visualization of the interstructure obtained via STATIS by analyzing the contribution of all 14 data tables to pairs of principal components–PC1 and PC2 (**A**), PC2 and PC3 (**B**). Principal components are ordered according to the magnitude of the associated eigenvalues (marked by darker bars in the inlays). The projection of the vector associated with each data table on the respective principal components indicates the contribution to the corresponding principal component.

To understand the effect of different accessions on the compromise, we subsequently investigated the table interstructure by quantifying the contribution of the tables to the first three principal components (denoted by PC1, PC2, and PC3) of the compromise space. By jointly considering primary and secondary metabolites, the data tables of 282, 156 (two *F*. *chiloensis* accessions) and 591 (*F*. *mochata*) contribute the most of the PC1 while showing minor contribution to PC2 (Fig. 4). By contrast, all domesticated strawberry together with 595 (*F*. *chiloensis* accession) and 196, 660, 191 (*F*. *vesca* accessions) data sets show reciprocal contribution to PC2. Finally, when comparing the contribution to PC2 and PC3, we observe a reciprocal contribution to PC2 of the *F*. *vesca* accessions (196, 191, 660), *F*. *mochata* (591), *F*. *chiloensis* accessions (156 and 282) on one side, and all domesticated accessions (except for 595) on the other (Fig. 4B). This observation may be due to the fact that the wild octoploid specie *F*. *chiloensis* is one of the two accessions of strawberry that were hybridized to develop the domesticated octoploid strawberry^54–56^. The observed differences in the considered *F*. *chiloensis* accessions (named as 156, 282, and 595) in the contribution to PC1 and PC2 may be attributed to the fact that the ripe fruit from the two subspecies 282 and 156 are white, while for the subspecies 595, it is red (Fig. 1).

When considering secondary metabolites, the interstructure showed similar picture (Supplementary Fig. S4). On the other hand, when considering only primary metabolites, the scenario changes (Supplementary Fig. S4), which is again consistent with the fact that primary metabolite of domesticated and wild accessions are very similar.

### Differential correlation-based network analysis of metabolite data

The identification of metabolites underlying metabolic differentiation solely based on individual changes largely neglects their inter-dependencies. Such relationships represent coordinated behaviors that can be captured (partly, due to the linear nature) in correlation-based networks. Metabolites undergoing major changes in metabolic co-regulation are expected to exhibit differential connectivity (DC) across networks, and can be employed in understanding of metabolic differentiation in the process of domestication of strawberry. Here we employed a differential correlation-based network approach to compare domesticated (D) and wild (W) accessions. Each of the two metabolic networks, denoted as network D and network W, comprises metabolite-metabolite associations based on the significance of Pearson correlation coefficients, adjusted using a false discovery rate (FDR) of 0.05. In addition, we conducted the analysis separately for the three maturation stages (i.e. green, white and red fruits) aiming at understanding whether such changes are stable as the developmental process unfolds. After quality checks (see Methods), a total of 39 primary metabolites (GC-MS) and 81 secondary metabolites (LC-MS) were used in the differential correlation-based network approach.

**Figure 5:**
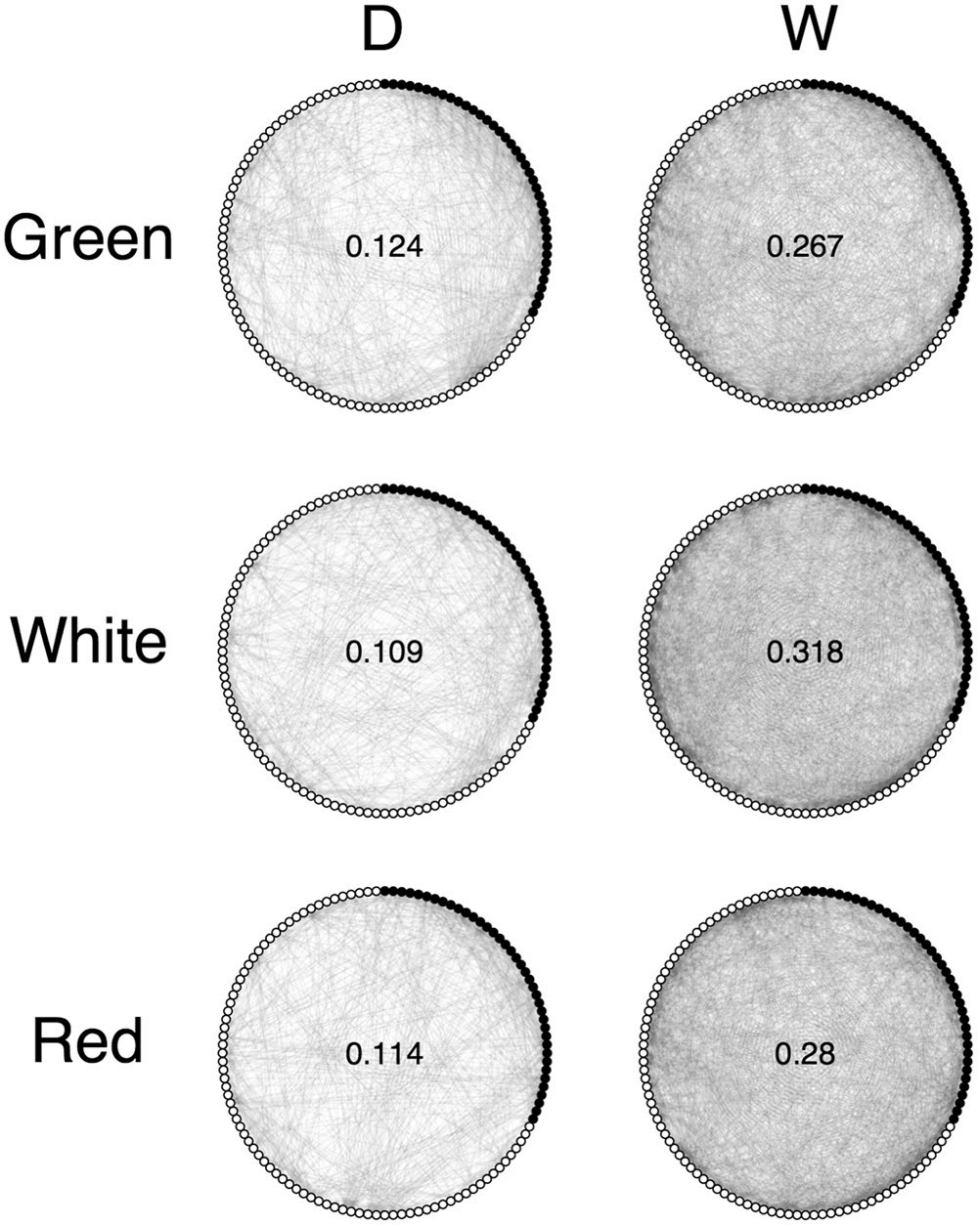
Comparative network analysis using domesticated (D) and wild (W) strawberry varieties. For every maturation stage (green, white and red), the correlation-based metabolic network from D was compared to that from W. Metabolic networks were re-constructed from significant metabolite-metabolite Pearson correlation coefficients (FDR of 0.05). The values shown inside the networks are the corresponding densities. Filled circles represent primary metabolites (GC-MS) and open circles represent secondary metabolites (LC-MS).

We first observed that the resulting metabolic networks were systematically denser for W compared to D. Density denotes the proportion of significant correlation between pair of metabolites (i.e. edges present in the network) from the total number of metabolite pairs, and it has been suggested to reflect the level of regulation in the network. Network D has densities of 0.124, 0.109 and 0.114 when green, white or red stages are considered, respectively. The observation about density result in the values of 0.267, 0.318 and 0.280 for the network W for the three progressive ripening stages, respectively (Fig. 5). Interestingly, when primary and secondary metabolites were considered separately, we noticed the higher density in network W is strongly driven by secondary metabolites (Supplementary Fig. S5).

**Figure 6:**
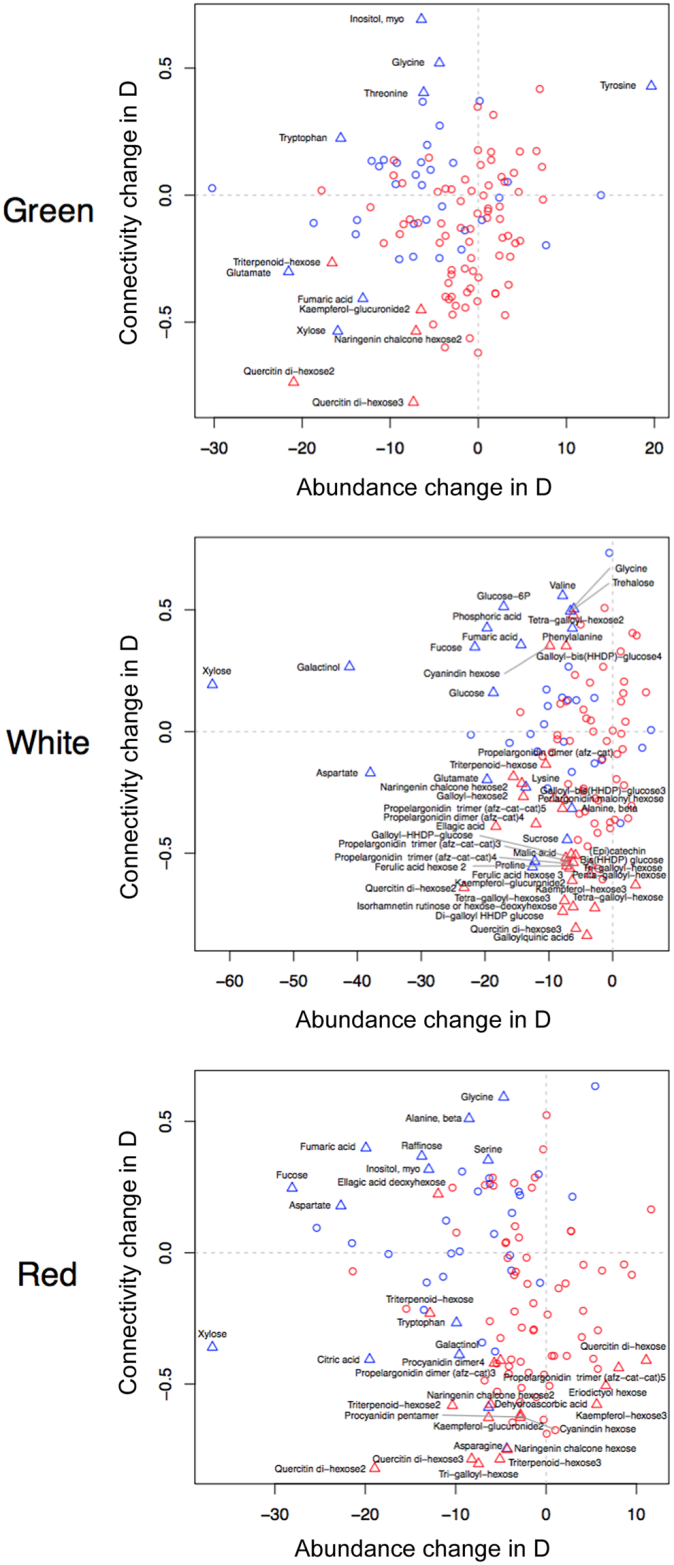
Analysis of differential connectivity (DC). Connectivity change in D and abundance change in D are shown across all three maturation stages (green, white and red, encoded as G, W and R respectively), depicted. Blue and red symbols represent primary metabolites (GC-MS) and secondary metabolites (LC-MS), respectively. Triangles highlight metabolites with significant DC and abundance change (FDR of 0.05).

For every metabolite, we determined its DC by subtracting the number of edges incident on the corresponding node in the network W to that in network D. To understand how DC relates to the difference in the levels of each metabolite, we also performed multiple *t*-tests to determine differences in relative abundance in D and W. *P*-values relative to DC were computed from a metabolite-specific permutation scheme (see Methods), and both lists of *P*-values (DC and *t*) were separately adjusted using a FDR of 0.05. Finally, we combined the two metrics to screen metabolites that simultaneously display significant changes in co-regulation and relative abundance (Fig. 6). A total of four possibilities fall into this scenario, i.e. any given metabolite in D, relatively to W, exhibits *i*) gain of connectivity and increased abundance (top-right quadrant), *ii*) gain of connectivity and decreased abundance (top-left quadrant), *iii*) loss of connectivity and decreased abundance (bottom-left quadrant), or *iv*) loss of connectivity and increased abundance (bottom-right quadrant).

We have identified a total of 13, 40 and 32 significant changes in green, white and red strawberries (Supplementary Table S1). More specifically, we found that triterpenoid-hexose, quercitin di-hexose (isomers 2 and 3), tri-galloyl-hexose and naringenin chalcone hexose (isomer 2) displayed a loss of connectivity and decreased relative abundances in all three stages. Xylose exhibits a decreased relative abundance across all conditions, but surprisingly changes from a state of loss of connectivity (green) to a gain (white) and back to a loss of connectivity (red). Galactinol, another sugar-alcohol, exhibits the same patterns in white and red strawberries. Fumaric acid also exhibits decreased relative abundance in all three stages, changing from a loss of connectivity (green) to a gain of connectivity (in both white and red stages). Glutamate displays decreased relative abundance and loss of connectivity in green and white strawberries, accompanied by proline in the latter stage. Putrescine was not found among metabolites significant for both metrics; however, glutamate, proline and putrescine were all significantly more abundant in W compared to D in all stages (Supplementary Table S1). Kaempferol-hexose (isomer 3) displays increased relative abundance and loss of connectivity in white and red stages. Other compounds that are more abundant in domesticated varieties include tyrosine, with a gain of connectivity in green strawberries, and quercitin di-hexose (isomer 1), propelargodinin trimer (isomer 5) and eriodictyol hexose with a loss of connectivity in red strawberries. Overall, there is an association between primary and secondary metabolites and gain and loss of connectivity, respectively (Pearson’s ${\chi }^{2}$ with Yate’s continuity correction of 7.11, 9.07 and 3.97 with *P* of 0.007, 0.002 and 0.046 for green, white and red strawberries, respectively). The investigated categorization of metabolites based on changes in their relative abundance and connectivity in the network demonstrates that the ripening process in wild and domesticated accessions is controlled by different mechanisms which shape the abundance of selected metabolites. To this end, we ask if there are metabolites which show differential behavior between the populations of wild and domesticated accessions.

### Multiple metabolic features as biochemical markers

Additionally, a mixed-effect model was constructed to identify metabolites that highly differ between the domestication status of domesticated and wild accessions (see Methods). We identify a total of 29 metabolites that exhibited significant domestication status effect (Supplementary Table S2), demonstrating that they can serve as biomarkers for the difference between domesticated and wild accessions. We observed that 26 of them were secondary metabolites, 7 classified as galloyl derivatives, four propelargonidins, six flavonols/flavanols, three terpenoid derivatives, three ellagitannins and one flavanone (Supplementary Table S4 and Figs S6 and S7). By contrast and in agreement with the correlation-based networks, only three primary metabolites changed significantly (named as glutamate, putrescine, and proline). The latter finding is also in line with STATIS analysis in which not major differences in primary metabolism.

## Discussion

Phenotypic evaluation of the collected germplasm is a prerequisite for germplasm prioritization or breeding program design but it is not straightforward and often indirect. The availability of high-throughput technologies such as metabolomics, facilitates dissecting a phenotype at the metabolic level, potentially enables more precise evaluation of sensorial, nutritional qualities, and disease resistance traits than those of traditionally used assessments such as agronomic and commercial traits. Metabolites themselves may also form the end phenotype which could be directly selected for (i.e. the presence of defense compounds maybe used to select for disease resistance).

The aims of the present study were to provide a comparative metabolome analysis of seven domesticated and seven wild strawberry accessions, in particular primary and secondary metabolism during fruit ripening and also, to determine if these data have the power to distinguish between accessions. If the latter is feasible, we were interested in identifying the metabolites which contribute most to the distinction between the two groups of accessions, which can be used to increase the narrow genetic base of domesticated strawberry.

The changes on composition of primary and secondary metabolites during strawberry fruit ripening have been compared based on STATIS, an extension of classical PCA, which allows us to jointly analyze the high-dimensional multivariate data sets. STATIS is able to distinguish and classify together the accessions from the same species as shown for *F*. *vesca* and *F*. *chiloensis* accessions. (Sub)species-specific differentiation was mainly due to qualitative and quantitative differences in accumulation of secondary metabolites, reflecting variation in the activities and substrates specificities of corresponding enzymes. Thus, it may be expected that the observed metabolic differences form the basis for species-specific differences in important traits (i.e. resistance, aroma, color, nutritional quality) and opens up possibilities to breed for novel these novel traits in cultivated domesticated strawberry backgrounds. To strengthen this claim, we conducted a clustering analysis of the wild and domesticated accessions based on different sets of other traits (see Supplementary Fig. S8), demonstrating correspondence to the clustering based on secondary metabolites.

In this vein, this differentiation was not observed for domesticated accessions. This observation may be due to the domestication and selection history of the accessions studied. Indeed, although the selected domesticated accessions are from different breeding programs (see Methods section), all of them have been selected for similar breeding purposes and therefore may have been exposed to similar selection history, including selection for desirable traits during breeding. Therefore, the differences among compound classes and single metabolites are strongly determined by the accessions group, indicating that genetic differences between accessions are reflected in metabolic differences. This may be due to differences in the regulation of metabolic pathways, differences in the activity of key enzymes determining the flux through the pathway, or the activity or substrate specificity of specific modifying enzymes.

In general, correlation network analysis suggests that metabolism during strawberry fruit ripening is highly coordinated. We observed that the metabolic networks in wild accessions were denser than the networks from the domesticated ones in all three stages of development. However, when primary and secondary metabolites were separately analyzed, the latter were largely responsible for the difference, suggesting that the process of domestication of strawberry lead to a general deregulation of the secondary metabolism and maintenance of the core processes that include primary metabolites. This observation was supported by the significant predominance of primary and secondary metabolites among metabolites displaying gain and loss of connectivity in the domesticated varieties, respectively, in all three stages of development. Altogether, these findings are consistent with the selective pressure of specific environmental stresses, elicitors of secondary metabolism, being no longer exerted in the process of strawberry domestication. Furthermore, we note that the difference of density between the two networks does not affect the subsequent results from the differential connectivity analysis, as the reported metric (DC) is normalized for the maximum value in each case (see Methods). The differential connectivity approach highlights the changes in metabolic co-regulation underlying strawberry maturation by examining the differences between domesticated and wild accessions across each individual developmental stage. Following the leading attempts in potato^57^ and grapevine^58^, here we also consider that higher metabolic co-regulation reflects a prioritization of the affected metabolic pathways. Upon complementation with *t*-tests, we were then able to simultaneously identify changes in co-regulation and abundance between the two groups and the corresponding differences in the three developmental stages, for each metabolite. For example, while xylose is more abundant in wild accessions in all three stages, it is less co-regulated in domesticated varieties in green and red stages, but in between it becomes more tightly regulated in white domesticated stage. This might suggest that, in spite of no discernible change (stage-wise) in the difference (accession-wise) in abundance, the associated metabolic processes gained more relevance in the strawberry ripening, specifically at the white stage, by means of domestication. On the other end of the spectrum, compounds such as tyrosine and procyanidin trimer (isomer 5), associated with tighter co-regulation and higher abundance in domesticated varieties, could represent those that were selected or co-selected for. However, we still can identify metabolites that are more tightly co-regulated in domesticated varieties and still less abundant, compared to wild accessions. These too could be favorable traits, including sugar-containing ellagitannins such as ellagic acid deoxyhexose, galloyl−bis(HHDP)−glucose found to be particularly abundant during early achene development^13^. Interestingly, important secondary metabolites playing a role in plant adaptation to the environment^59^ were associated with tighter co-regulation and higher abundance in wild accessions, such as triterpenoid-hexose, quercitin di-hexose [isomers 2 and 3], tri-galloyl-hexose, kaempferol-hexose and naringenin chalcone hexose. Moreover, these metabolites were identified as the most contribute to distinguish between the wild and domesticated accessions, being clear candidate to be markers for selection.

Correlation-based network analysis highlighted an extent of connectivity, building accession/stage-specific metabolite nodules. The metabolites allowing this degree of connectivity are probably different across the accessions, but this type of evidence suggests some common mechanism among domesticated and wild strawberry fruit at the basis of metabolic regulation involving a high connectivity of primary metabolites mainly in early developmental stages. One of the disadvantages of using degree of connectivity, is that it is not possible to distinguish direct or indirect interactions between metabolites, since a third metabolite can be responsible of this connectivity.

### Conclusions

These results of this study clearly demonstrate that there is a large metabolic variation present in the strawberry germplasm collection analyzed. Our analysis based on STATIS indicates that the wild and domesticated strawberry accessions differ with respect to the secondary metabolites. Moreover, STATIS was able to distinguish and classify together the accessions from the same species. By applying a mixed-effect model we were able to identify the metabolites which contribute most to the distinction between wild and domesticated strawberry accessions, which can be used to increase the narrow genetic based of domesticated strawberry. We also observed that the relative abundance of the analyzed metabolites varied greatly among fruits of wild accessions, demonstrating the potential of these germplasm collection for genetic improvement of metabolic traits, although the success of the breeding program is strictly related to combining attitude of the different parents.

The correlation network analysis demonstrated that overall, the secondary metabolism of wild strawberry accessions was more tightly coordinated than the domesticated accessions, which suggests that the domestication process lead to a deregulation of the secondary metabolism. The differential connectivity analysis, on the other hand, shed light on individual metabolites that exhibit a significant change of coordination with other metabolites, which in the latter case translates into the prioritization of key metabolic processes. In association with the differences in relative abundance then, the two metrics helped in concurrently determining how levels of metabolite and their control change across the three stages of maturation described herein.

These analyses formed are an effective tool to understand metabolic variation between strawberry accession, and may serve as a bridge to understanding the manifestation of economically important traits. For this reason, this type of experiment can be considered important for the production of new genotypes with improved specific metabolic traits, and for the identification of accessions that can perform in the same way as the most useful parents. A combined use of high throughput genotyping and metabolomics techniques should provide a new avenue to discover novel associations between allele frequency and metabolic variation in strawberry populations. Such integration will enable us to conduct targeted molecular breeding of the metabolites that contribute to the adaptive or nutritive value of new improved strawberry varieties.

## Methods

### Plant materials and growth conditions

A panel of seven *Fragaria x ananassa* accessions from different breeding programs were chosen to represent a large proportion of commercial strawberry acreage in South Spain from both public (Amiga, Santa Clara, Fuentepina, Fontanilla) and private (Candonga) breeding programs. Additionally, North American accessions were added to enhance the range of diversity (Camarosa and Benicia). Also, seven wild strawberry accessions from different origins were included in this study (Fig. 1; three *F*. *chiloensis*, 156, 282, 595; three *F*. *vesca*, 196, 191, 660; *F*. *moschata*, 591).

All strawberry plants were grown and maintained under glasshouse conditions (IFAPA, Spain). Fruits were harvest in three different developmental and ripening stages corresponding to green (G), white (W) and ripe (R) (Fig. 1). These fruits were collected during later March–early June. Metabolome analysis was performed in three separate pools of 13–25 fruits each. Each pool was from three different plants. All fruits were frozen immediately in liquid nitrogen and kept at −80 °C until further analysis.

### Extraction, derivatization, and analysis of polar metabolites using GC-MS

Metabolites analysis by GC-MS was carried out as previously described^60^. The mass spectra were cross-referenced with those in the Golm Metabolome database^61^. Full documentation of metabolite profiling data acquisition and interpretation is provided in Supplementary Table S3.

### Extraction and analysis of semi-polar metabolites and UPLC-Orbitrap-MS/MS measurements

Metabolite extraction was performed as previously described^62^. In brief, 250 mg of each biological replicates was extracted with a cold mixture of methyl-*tert*-butyl-ether: methanol (3:1). To facilitate cell disruption samples were incubated in a cooled sonic bath for 10 min. The subsequent addition of a water:methanol (3:1) mixture to the extract resulted in a formation of two liquid phases. A fixed volume of polar phase was transferred to a fresh Eppendorf tube before concentrating it to dryness in a Speed-vac (Centrivac, Heraeus).

Chromatographic separation was performed using a Waters Acquity UPLC system equipped with a HSS T3 C_18_ reverse phase column (100 × 2.1 mm i.d., 1.8 µm particle size; Waters) which was operated at a temperature of 40 °C. The mobile phases consisted of 0.1% formic acid in water (Solvent A) and 0.1% formic acid in acetonitrile (Solvent B). The flow rate of the mobile phase was 400 µl/min, and 2 µl of sample was loaded per injection. The UPLC was connected to an Exactive Orbitrap (Thermo Fisher Scientific) via a heated electrospray source (Thermo Fisher Scientific). The spectra were recorded alternating between full-scan and all ion-fragmentation-scan modes, covering a mass range from 100 to 1500 *m/z*. The resolution was set to 35,000, and the maximum scan time was set to 250 ms. The sheath gas was set to a value of 50, while the auxiliary gas was set to 20. The transfer capillary temperature was set to 250 °C, while the heater temperature was adjusted to 350 °C. The spray voltage was fixed at 3 kV, with a capillary voltage and a skimmer voltage of 25 and 15 V, respectively. MS spectra were recorded from minute 0 to 19 of the UPLC gradient. The raw data acquired were processed using Xcalibur 2.1 software (Thermo-Fisher). Processing of chromatograms, peak detection, and integration were performed using REFINER MS 7.5 (GeneData; http://www.genedata.com). The obtained features (*m/z* at a certain retention time) were queried against the biological databases [KNApSAcK (http://kanaya.naist.jp/KNApSAcK/), Metabolome.Jp (http://www.metabolome.jp/), and KEGG (http://www.genome.jp/kegg/), Chemspider (http://www.chemspider.com/)]. The mass error for the database searches and the fragment assignments was always <2 parts per million (average value 0.8 ppm) resulting in confident elemental composition assignments. The MS/MS fragmentation of the metabolites was compared with candidate molecules found in databases, and verified with earlier literature on similar compounds, especially when the presence of the metabolite was reported in strawberry. Full documentation of metabolite profiling data acquisition and interpretation is provided in Supplementary Table S4.

### Overview of STATIS analysis

STATIS operates on the analysis of $K$ data sets (i.e. tables) denoted ${X}_{1},\cdots ,{X}_{K}$. In our analysis, we used data sets from $K=14$ strawberry accessions. The corresponding tables are combined in a block-matrix $X=[{X}_{1}|\cdots |{X}_{K}\,]$. In our case, each table includes the observations on $p$ variables (*i*.*e*., metabolites), corresponding to the columns, measured over ${t}_{1},\cdots ,\,{t}_{Kn}$ time points (*i*.*e*., stages), given by the rows. As previously stated, $n=3\,\,$is the same for all considered species, and $p$ was made the same by considering that an undetected metabolite has abundance of zero. Furthermore, we will use ${x}_{ij}^{l}$ to denote the (relative) content or abundance of the *j*^th^ metabolite in the *i*^th^ stage of the *l*^th^ accessions. Each tables was normalized and centered based on published recommendations^63,64^. Each row was assigned the same weight, so that all weights over rows sum of to 1 and are gathered in a so-called vector of masses ${\boldsymbol{m}}$.

We then compute the relationship between the rows of each matrix by determining

1 $${S}_{i}={X}_{i}{X}_{i}^{T},$$

the cross-product matrix for each of the $K$ tables. We then calculated $C$, a matrix whose entries are given by

2 $${c}_{ij}=\sum _{p=1}^{t}\sum _{q=1}^{t}{s}_{p,q}^{i}{s}_{p,q}^{j}.$$

The first eigenvector $\alpha$ of $C$, normalized to a unit vector, then yields the weights based on which the data tables are combined to obtain the compromise

3 $$S=\sum _{i=1}^{K}{\alpha }_{i}{S}_{i}.$$

Usually, matrices ${S}_{i}$ and ${S}_{j}$ are normalized such that the sum of squares of their elements equals 1. As both matrices are semidefinite, in this case, the inner product, ${c}_{ij}$, corresponds to the ${R}_{V}$ coefficient which is the cosine, taking values between 0 and 1, between the matrices ${S}_{i}$ and ${S}_{j}$ thus representing a well-defined measure of similarity^65^:

4 $${R}_{V}({S}_{k},{S}_{{k}^{\text{'}}})=\sum _{i}^{t}\sum _{j}^{t}{s}_{i,j,k}{s}_{i,j,{k}^{\text{'}}}$$

The second step of STATIS consists of a generalized eigenvalue decomposition of the compromise, whereby one can obtain the loadings of the variables.

### Mixed-effect models

Mixed-effect model was implemented for every metabolite separately by using the R package lme^66^. We used stage, replicate (rep), and population (pop) factors as fixed effects and the genotype (gen) as a random effect nested in population to model the level of each metabolite (meta). For completeness, we include the code snippet:

$${\rm{model}} < \,-{\rm{lmer}}({\rm{meta}} \sim {\rm{stage}}+{\rm{rep}}+{\rm{pop}}+(1|{\rm{pop}}:{\rm{geno}}),{\rm{data}}={\rm{dataall}})$$

Statistical testing for the fixed effects was conducted with the lmerTest function from the same package^67^. To support the identification of these metabolites, the MS and MS/MS spectrum have been included as Supplementary Fig. S7.

### Network-Based analysis

Metabolites displaying more than 20% of missing values were excluded from the analysis. Imputation of the remaining missing values was conducted using a Bayesian principal component analysis using five components (Stacklies *et al*., 2007- http://bioconductor.org/packages/release/bioc/html/pcaMethods.html). Metabolic networks (W for wild varieties and D for wild varieties) were re-constructed from metabolite-metabolite Pearson correlation coefficients (*r*) associated with FDR-adjusted *P*-values ($H0:r=0$), or *q*-values, using a significance level of 0.05. Density is considered as the number of observed edges divided by the total number of possible edges connecting all nodes, therefore ranging from 0 to 1.

The differential connectivity (DC), based on the resulting metabolic networks, was calculated as:

5 $$D{C}_{i}={K}_{i}^{D}-{K}_{i}^{W}$$

for every metabolite *i* with ${K}_{i}^{D}$ edges in network D and ${K}_{i}^{W}$ edges in network W, with both values normalized for maximum degree in the corresponding networks. Two-tailed Student’s *t* tests were performed to determine the *q*-values of the differences in metabolite levels between D and W ($H0:D=W$) using a significance level of 0.05. The *q*-values relative to DC were computed from empirical null distributions of DC after randomly permuting the W and D varieties 1,000 times, after^68^ and using a significance level of 0.05.

## Electronic supplementary material

Supplementery Information

Table S1

Table S2

Table S3

Table S4
