# Supplementary material for: Genetic diversity of strawberry germplasm using metabolomic biomarkers
Source: Sci Rep. 2018 Sep 26;8:14386. doi: 10.1038/s41598-018-32212-9 (PMC6158285; doi:10.1038/s41598-018-32212-9)
Supplement: Supplementary file 1 — Supplementery Information [file 41598_2018_32212_MOESM1_ESM.pdf]

Supplementary information

Genetic diversity of strawberry germplasm using metabolomic biomarkers

José G Vallarino, Francisco de Abreu e Lima, Carmen Soria, Hao Tong, Delphine M. Pott, Lothar Willmitzer, Alisdair R Fernie, Zoran Nikoloski, Sonia Osorio.

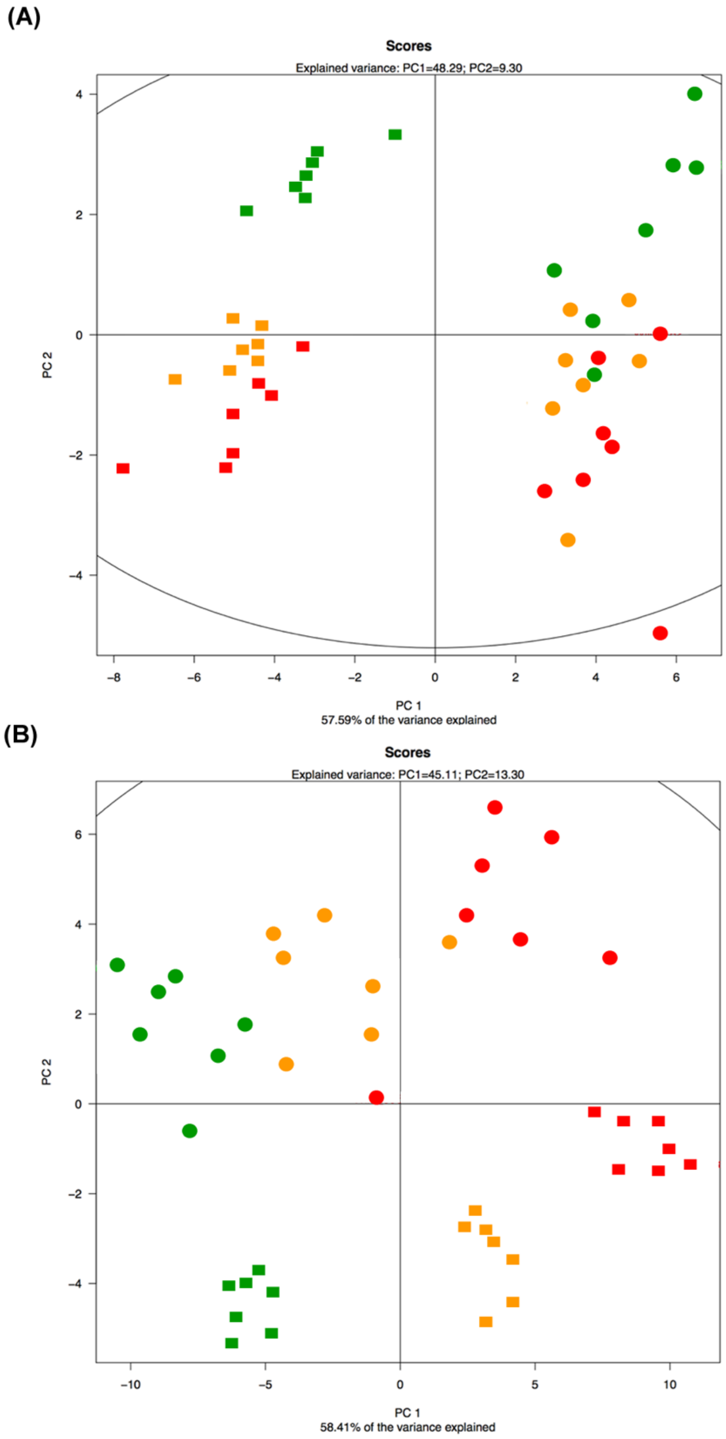

Supplementary Figure S1. Principal component analysis (PCA) of metabolites data of seven domesticated and seven wild strawberry accessions across different developmental and ripening fruit stages. PCAs of (A) primary metabolites and (B) polar secondary metabolites. Score plots of the PCA, domesticated (squares) and wild (circles) strawberry accessions. Green symbols, green stage; orange symbols, white stage; red symbols, red stage.

(A)

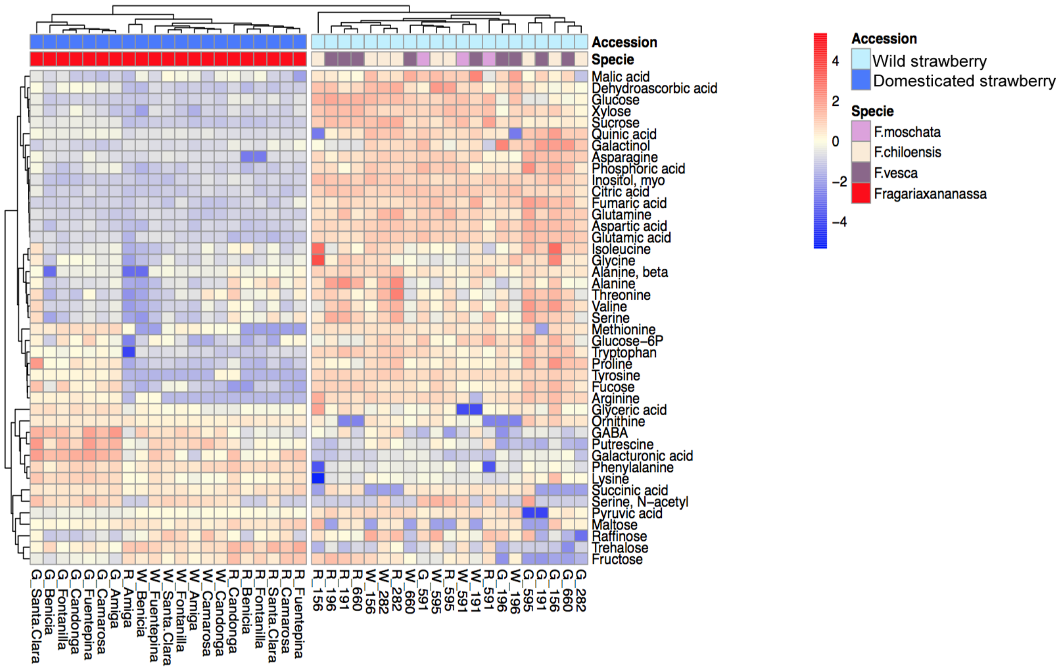

(B)

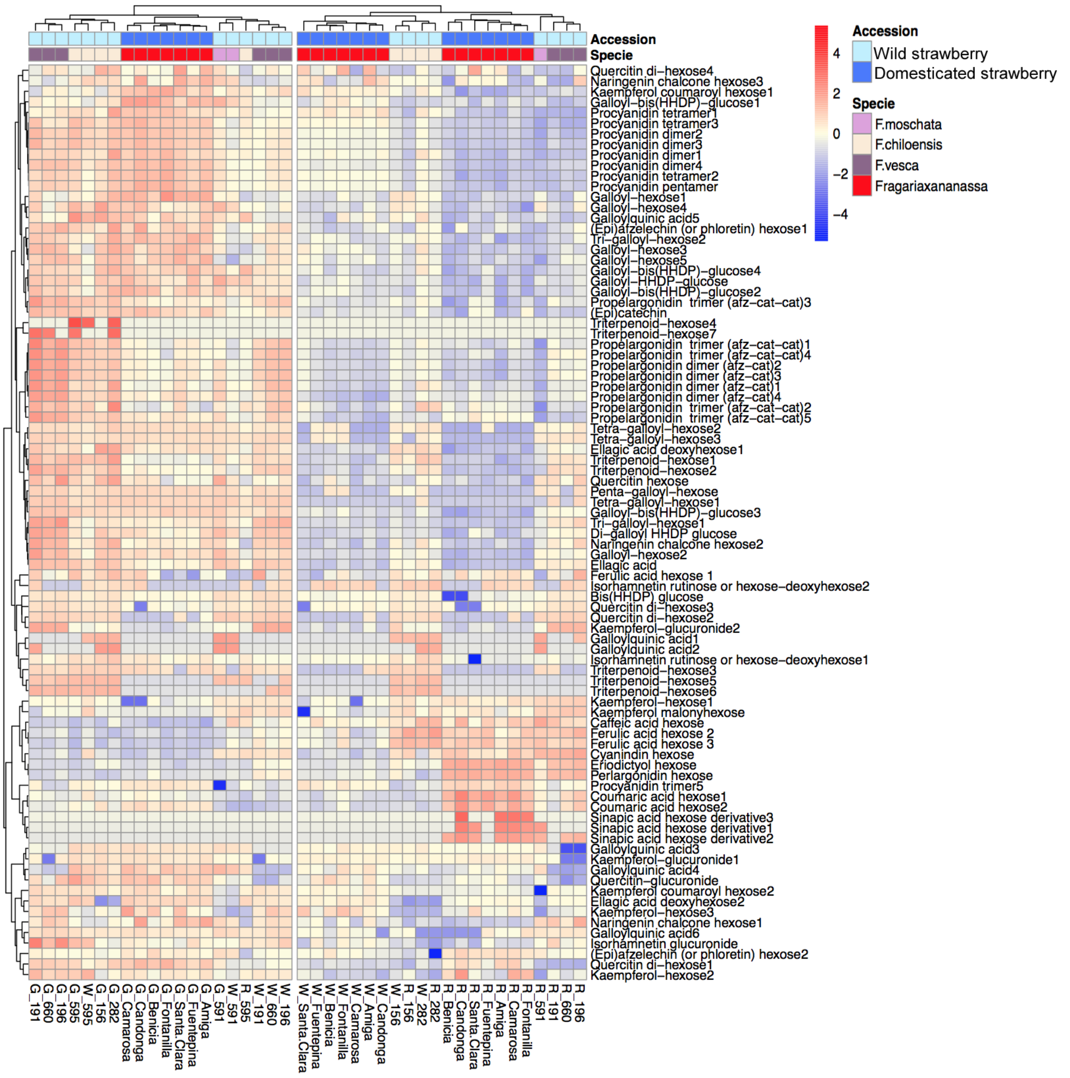

Supplementary Figure S2. (A) Heatmap of 43 primary metabolites, and (B) 87 polar secondary metabolites in seven domesticated and seven wild strawberry accessions across different developmental and ripening fruit stages. A color-coded matrix represents the mean values of the metabolite intensity in three biological replicates of strawberry accessions, which has been log2 transformed and mean-centered.

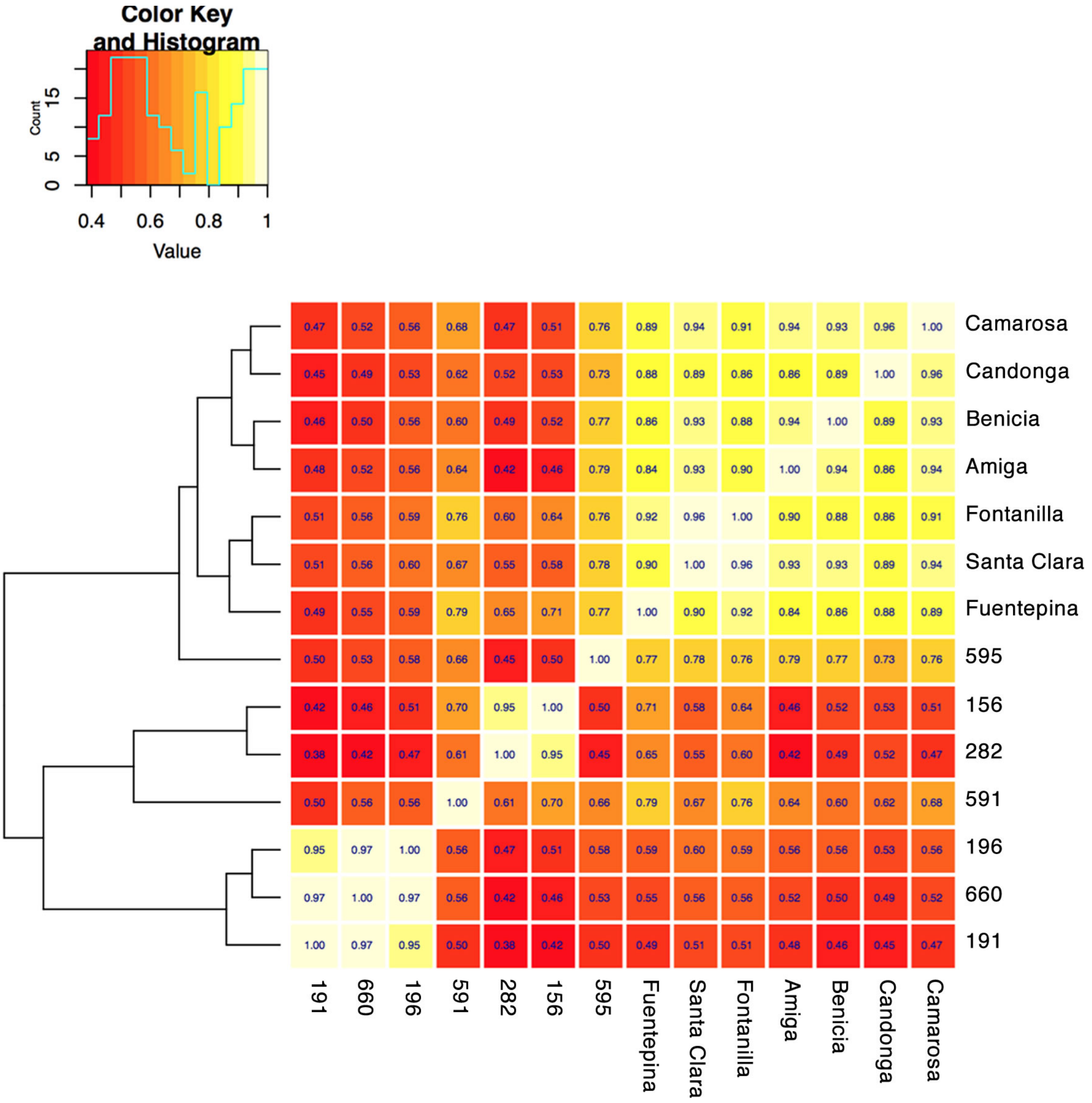

Supplementary Figure S3. Visualization of similarity of secondary metabolite based on hierarchical clustering and heatmap. Similarity between the tables from the different accessions is based on the Rv coefficient. A value of 1 for the Rv coefficient corresponds to equivalence (white), while a value of 0 indicates complete dissimilarity (dark red).

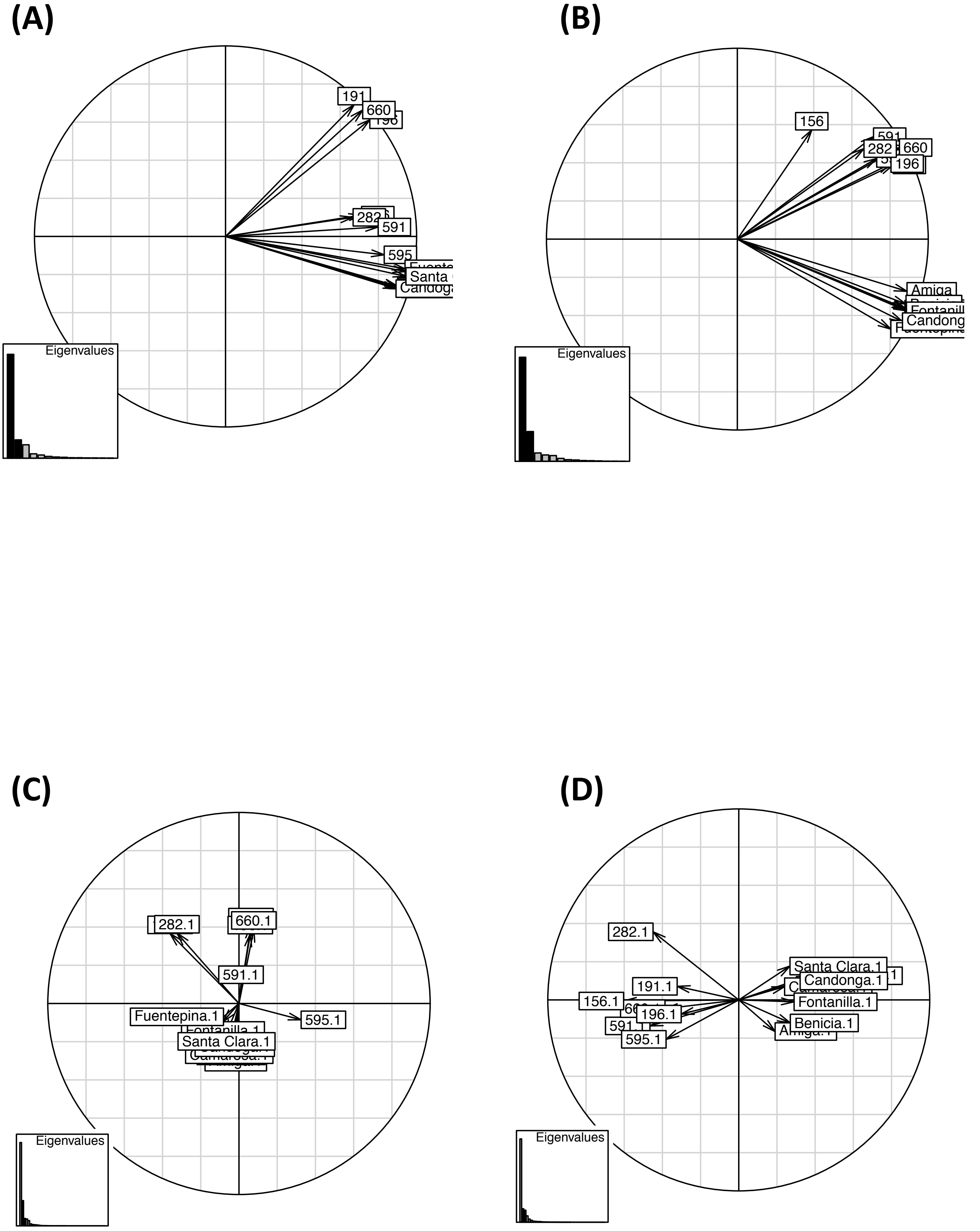

Supplementary Figure S4. Contribution of tables of accessions to PC1 and PC2 (A and B) and PC2 and PC3 (C and D) for secondary metabolites (A and C) and primary metabolites (B and D).

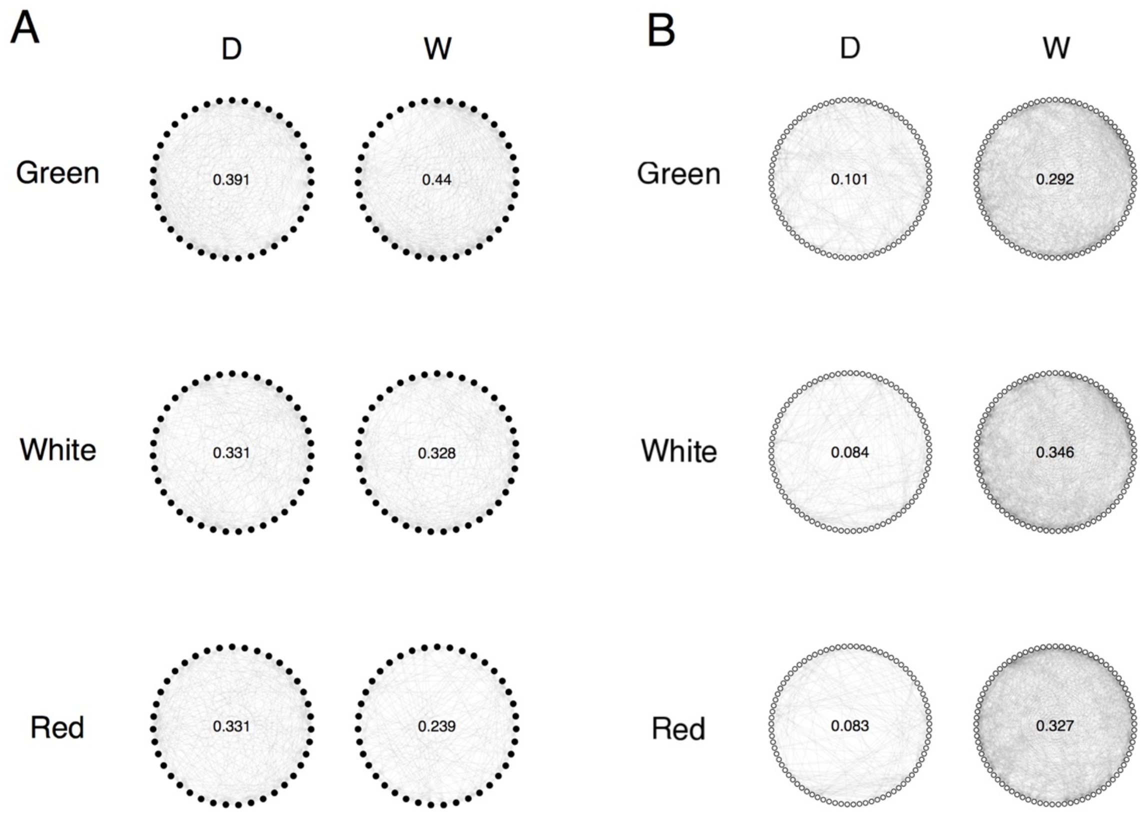

Supplementary Figure S5. Comparative network analysis using domesticated (D) and wild (W) strawberry varieties separately for each metabolomics platform. For every maturation stage (green, white and red), the correlation-based metabolic network from D was compared to that from W. The values shown inside the networks are the corresponding densities. (A) The comparative analysis using only primary metabolites (GC-MS) shows slight, unsystematic differences in density in the different stages. (B) The comparative analysis using only secondary metabolites (LC-MS) shows systematic differences in density that exceed more than 2-fold of D in all stages. Filled circles represent primary metabolites (GC-MS) and open circles represent secondary metabolites (LC-MS).

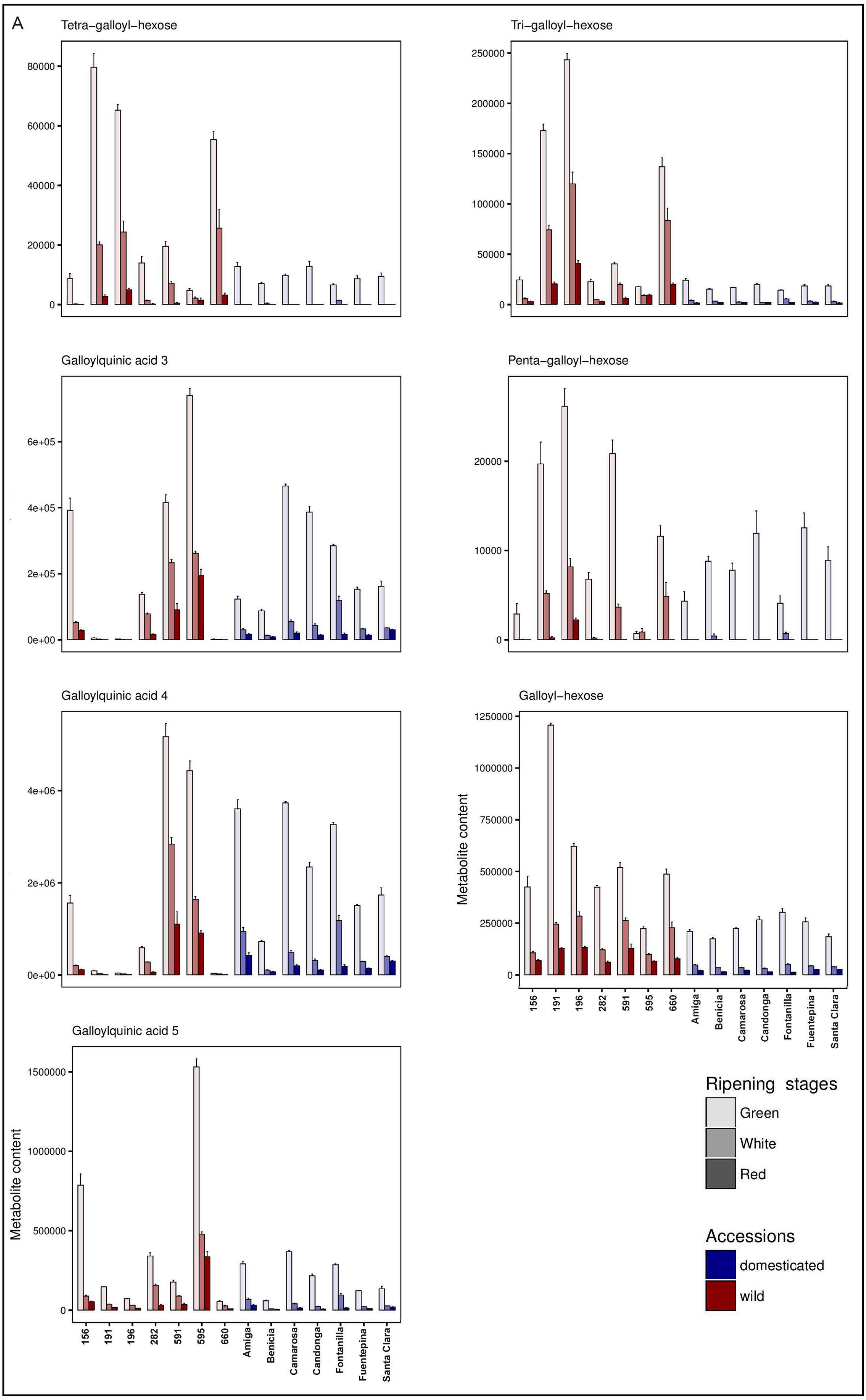

Supplementary Figure S6. Relative abundance in green, white and red fruits of the metabolites which exhibited significant population effect. (A) galloyl glucoses, (B) anthocyanins, (C) ellagitannins and ellagic acid precursor/derivative, (D) flavonols, flavanols, (E) flavanones, (F) propelargonidins, (G) terpenoid derivatives and H) primary metabolites.

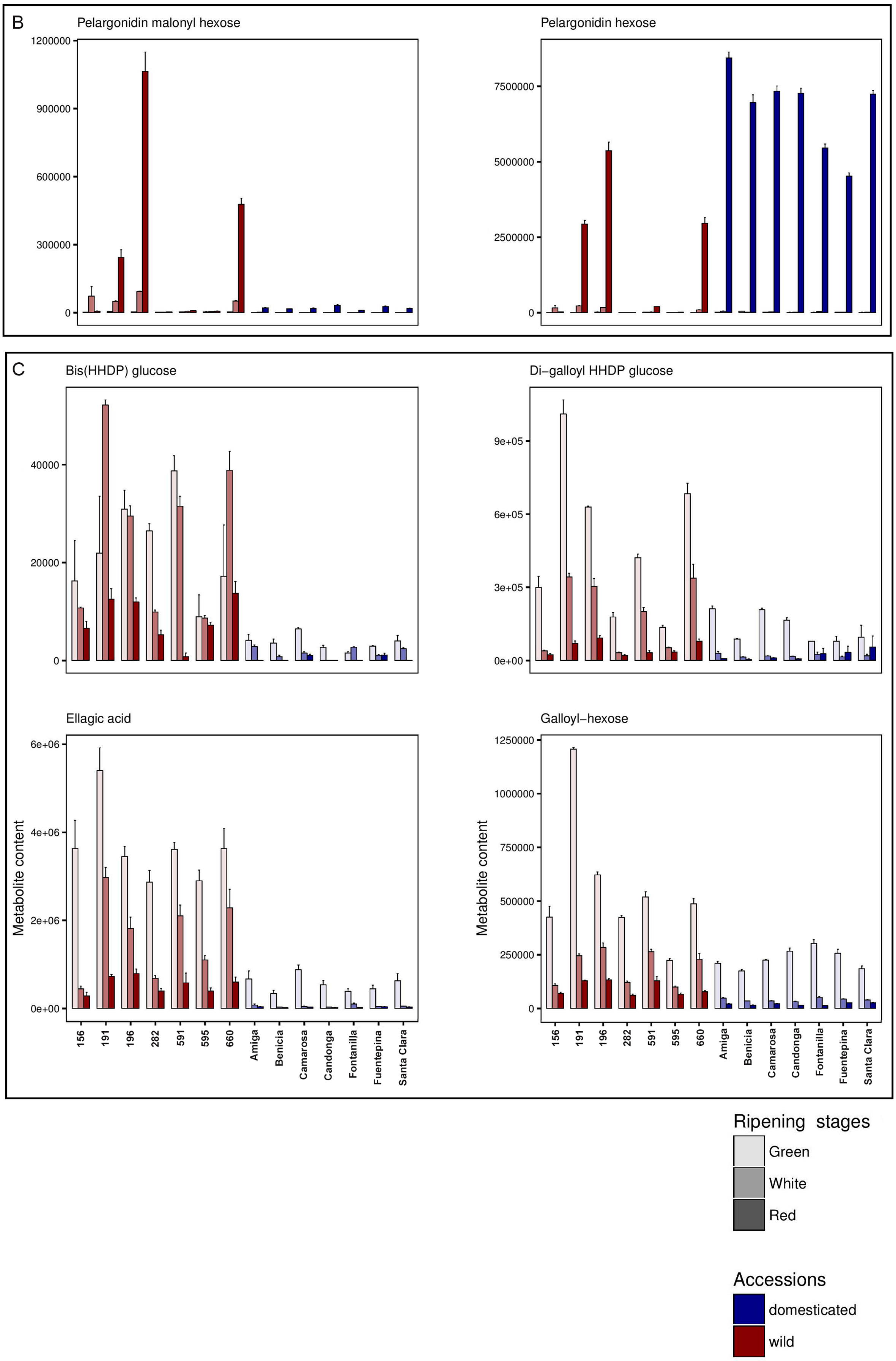

Supplementary Figure S6

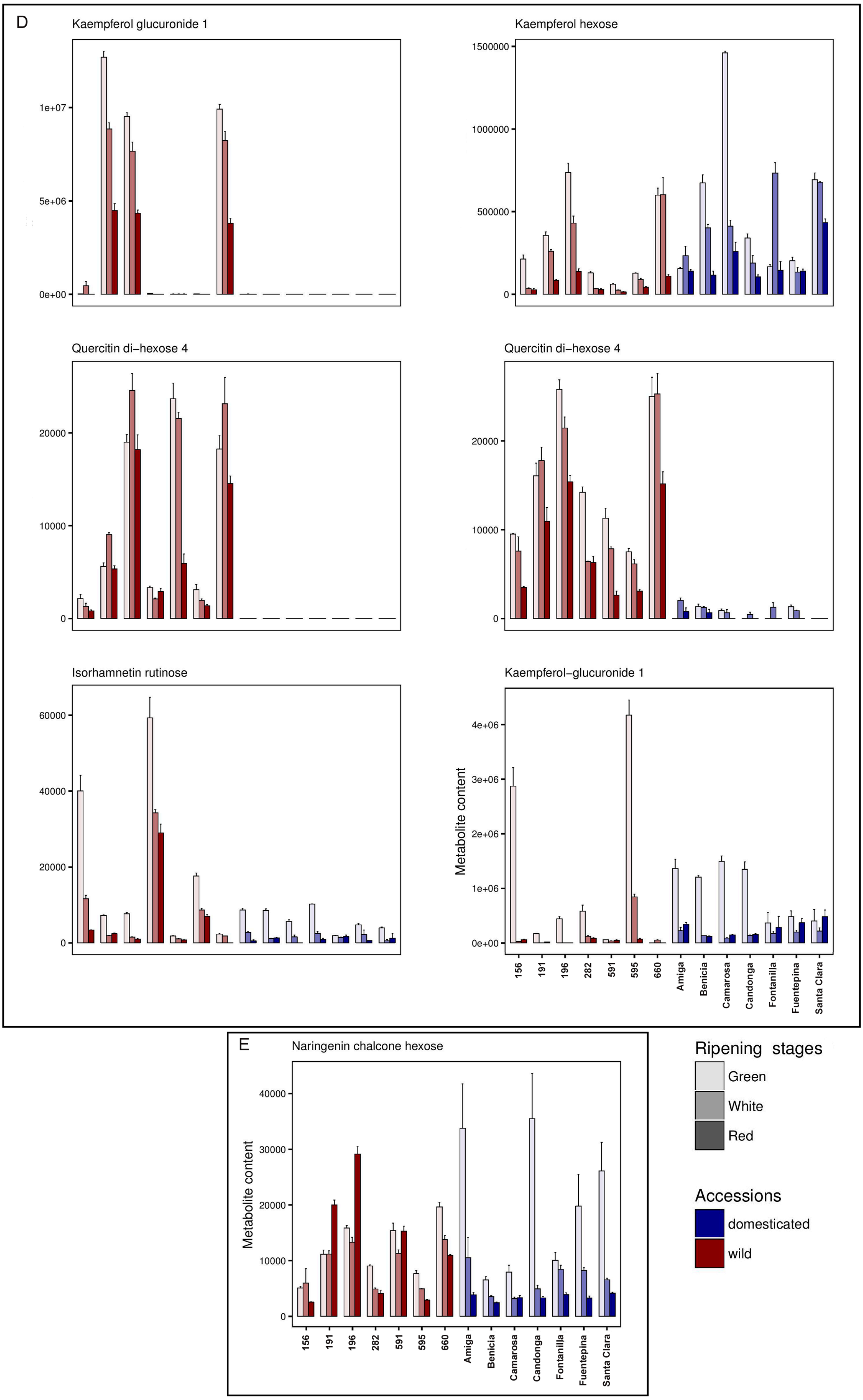

Supplementary Figure S6

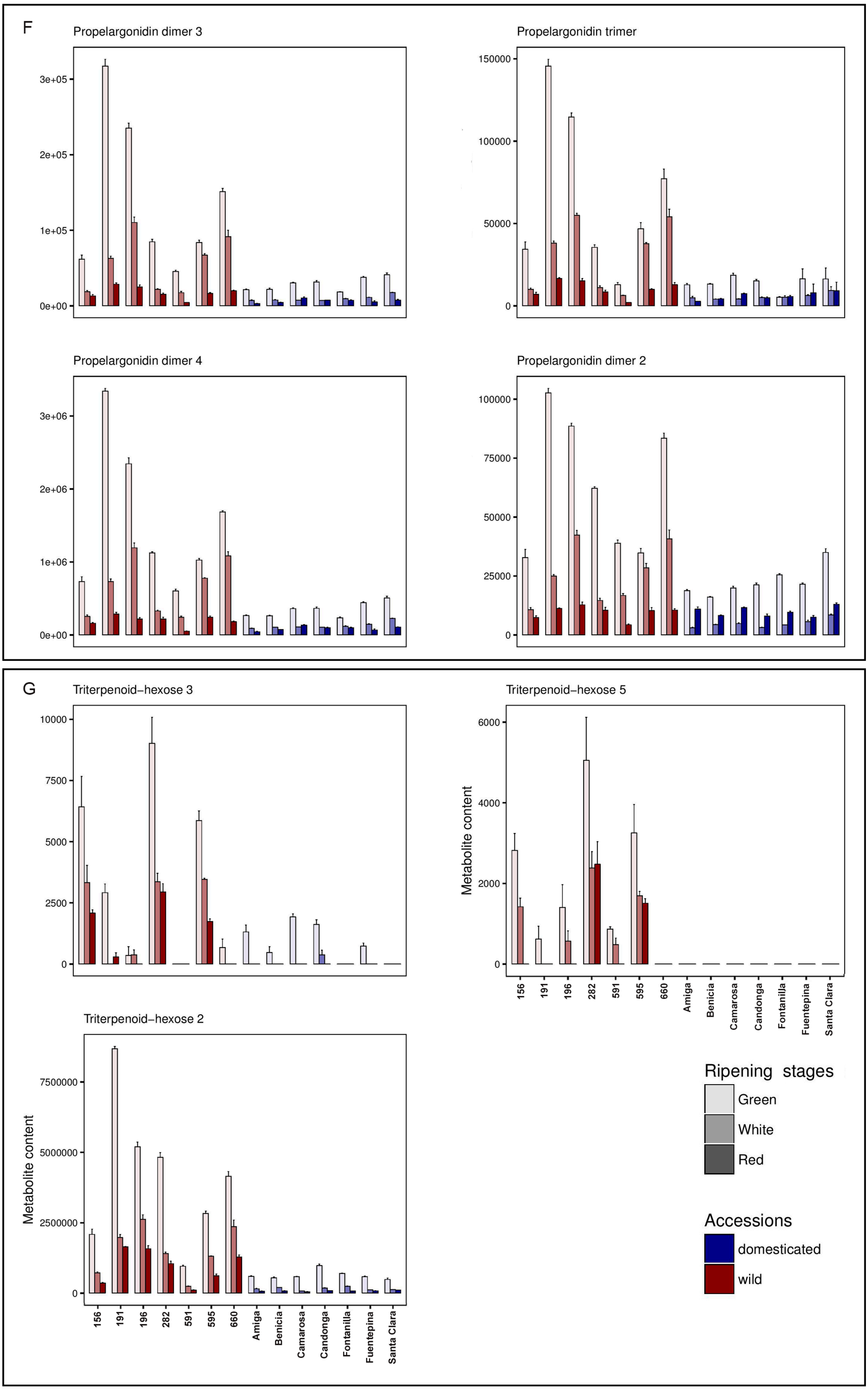

Supplementary Figure S6

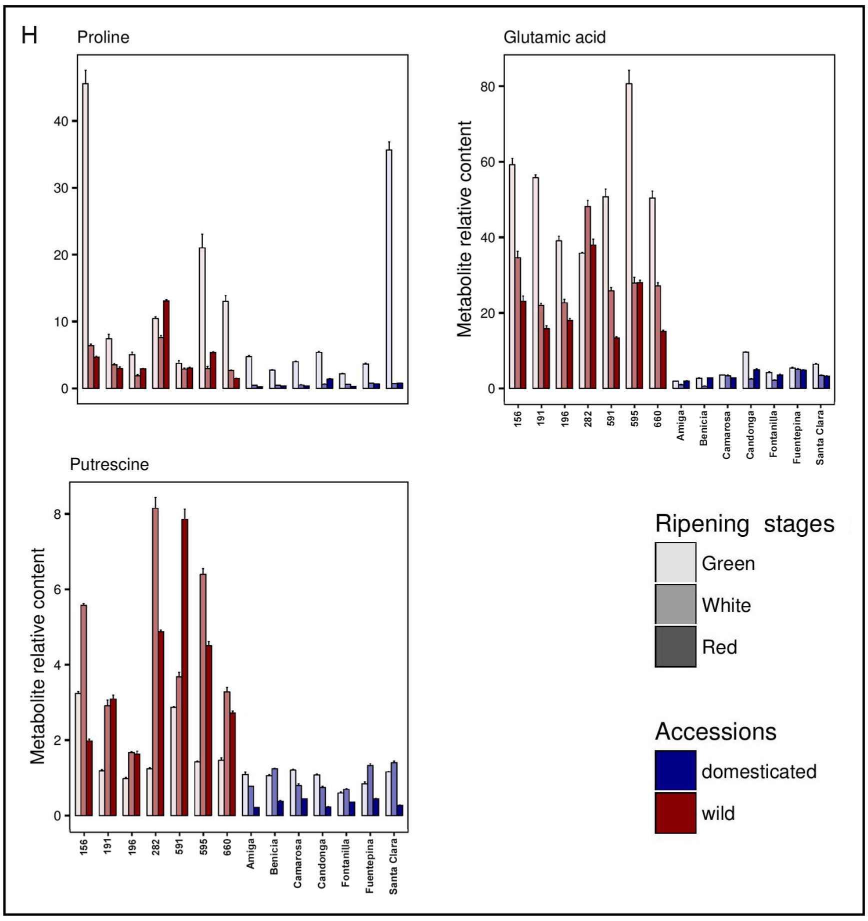

Supplementary Figure S6

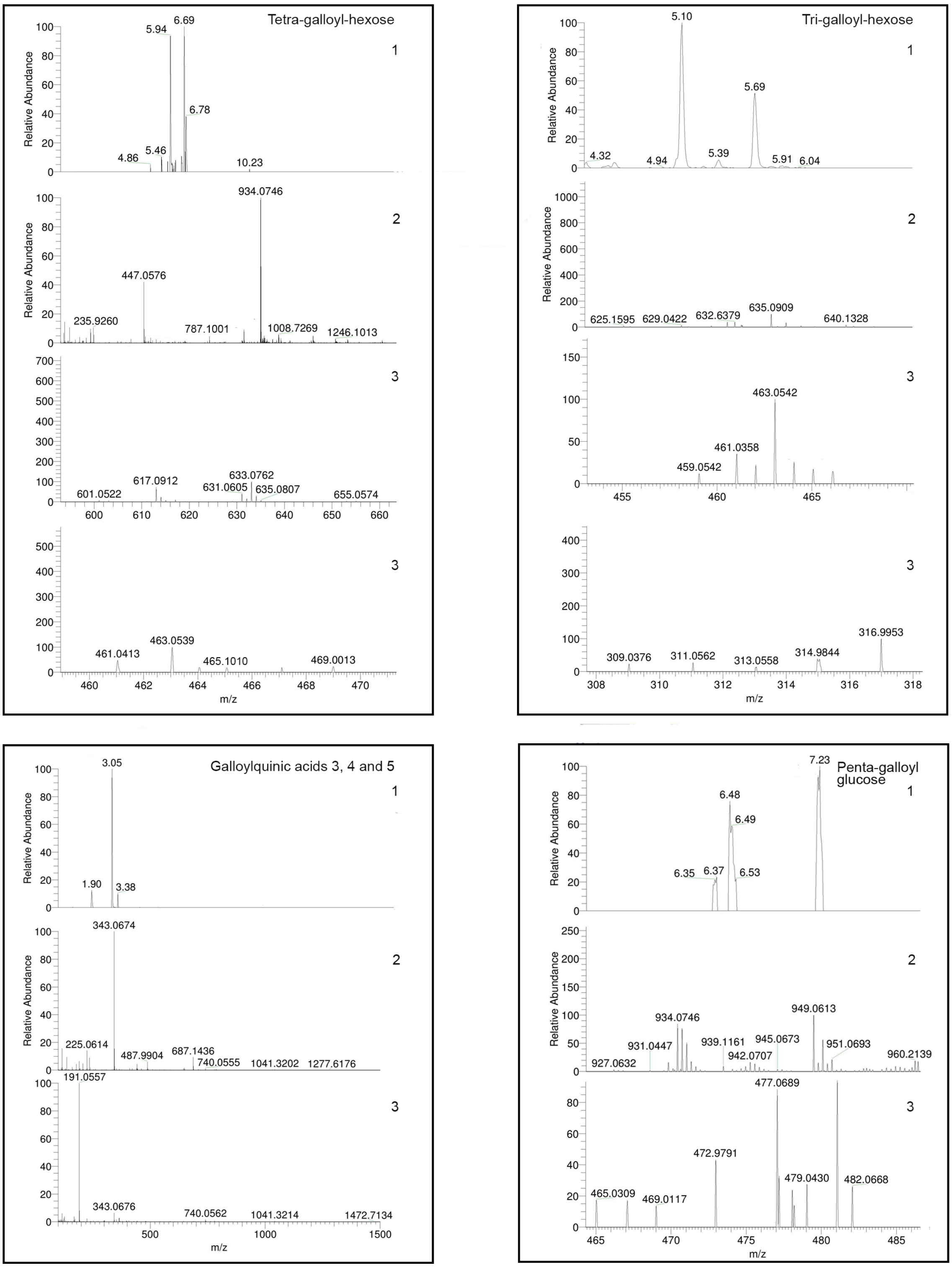

Supplementary Figure S7. Chromatograms (1), MS (2) and MS/MS (3) spectra of the metabolites which exhibited significant population effect. Spectra are shown for negative ionization mode, except where positive ionization mode is indicated. Spectra of different isomers for the same compound are only represented once.

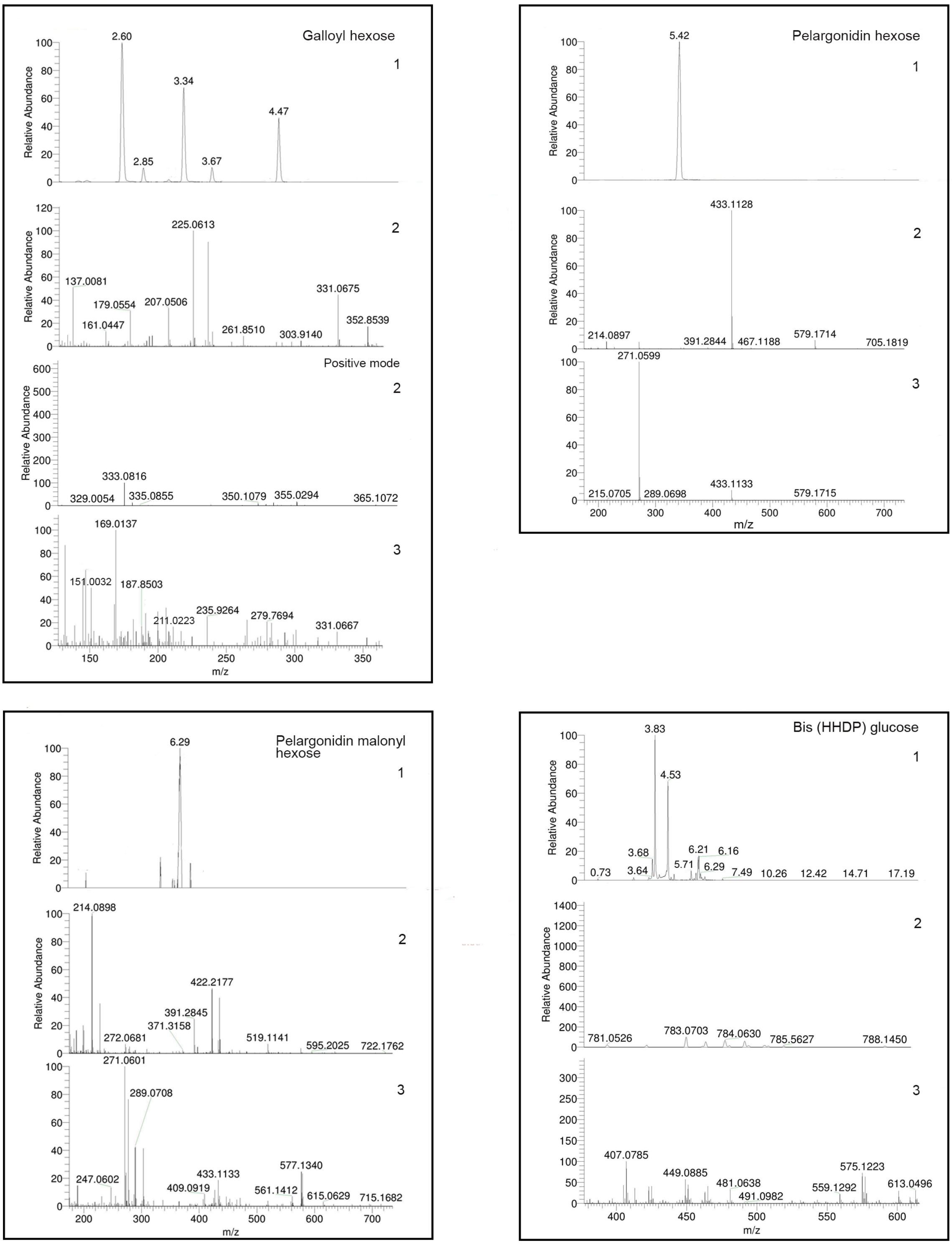

Supplementary Figure S7.

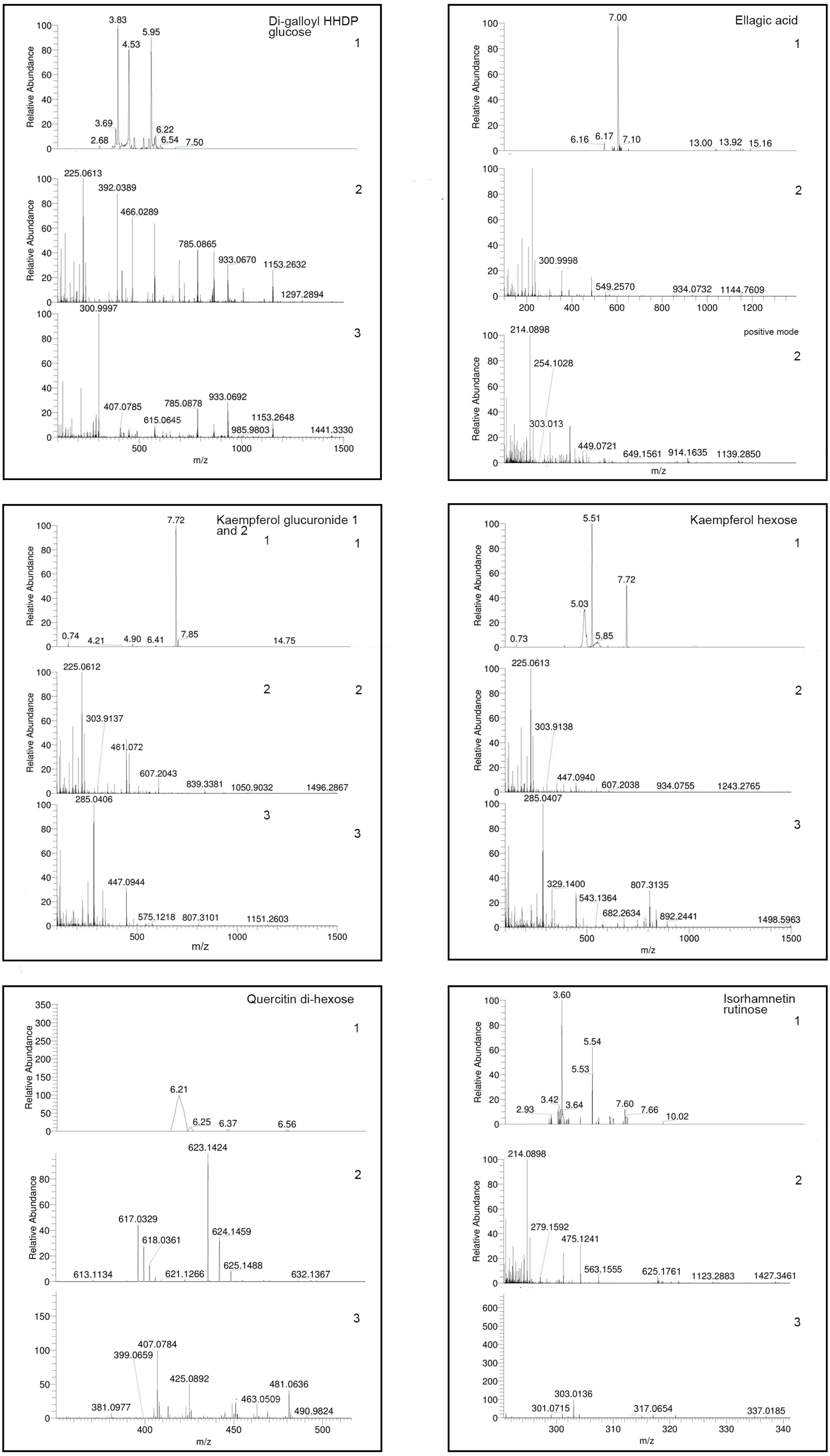

Supplementary Figure S7.

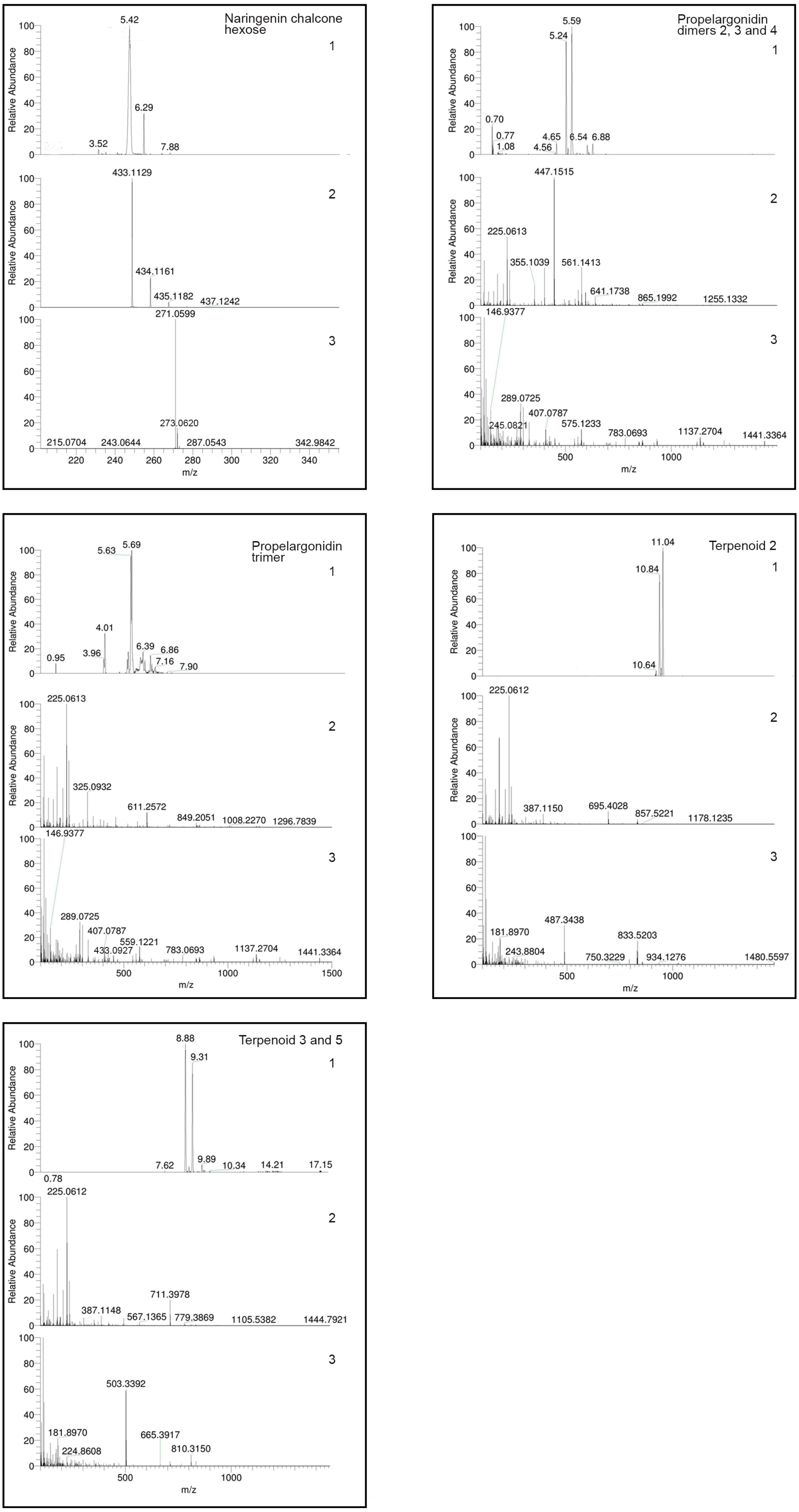

Supplementary Figure S7.

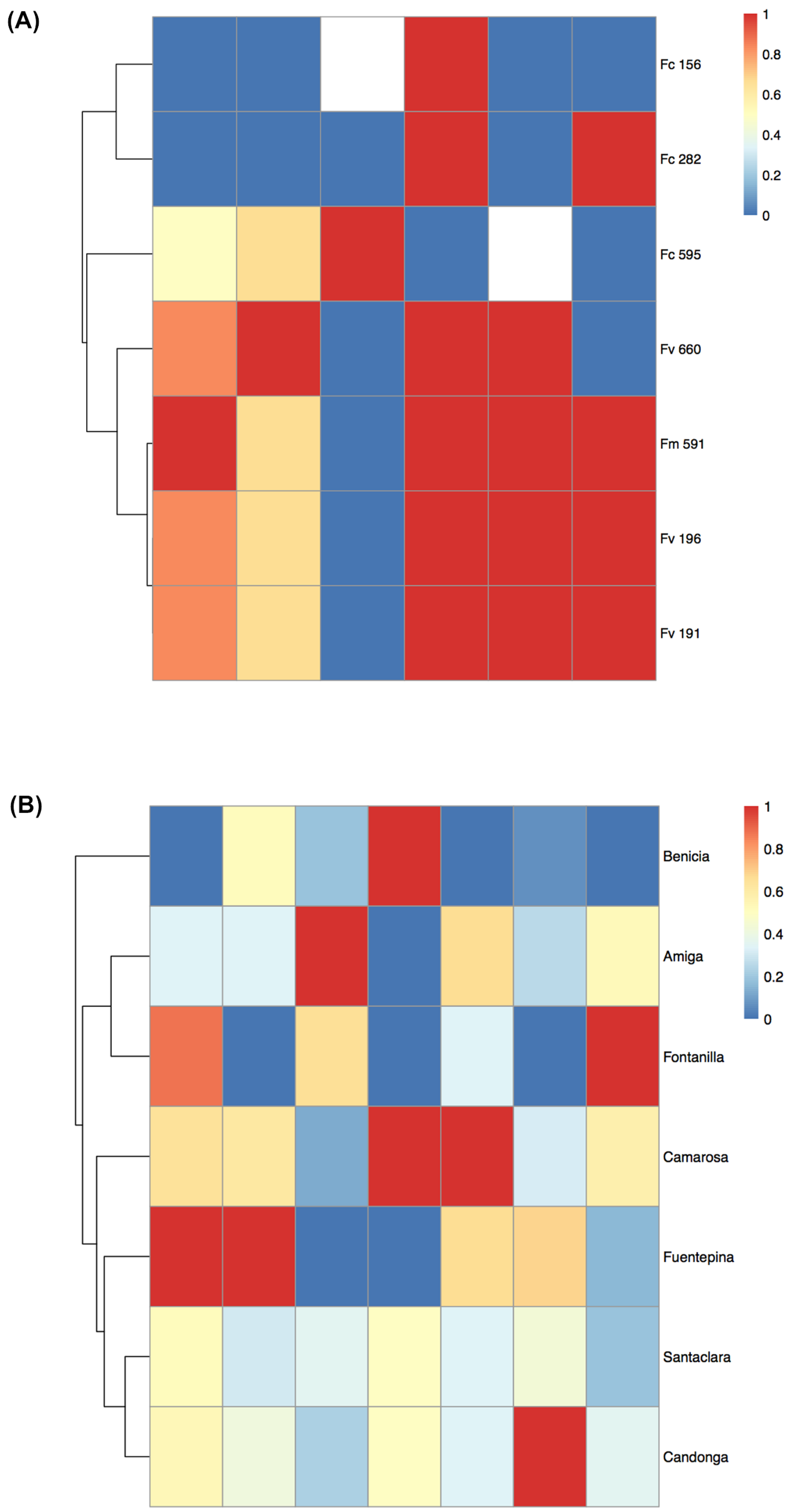

Supplementary Figure S8. Clustering of accessions based on complex traits**.** (A) wild accessions, based on growth habitat, plant vigor, plant density, number of flowers, color, and shape. (B) domesticated accessions, based on Brix, yield, plant diameter, weight, firmness, color, and shape. Heatmaps are based on correlation matrix after data normalization, and include a dendrogram from hierarchical clustering.
